# Low-cost tools for virtual reconstruction of traffic accident scenarios

**DOI:** 10.1016/j.heliyon.2024.e29709

**Published:** 2024-04-16

**Authors:** Juan Antonio Pérez, Gil Rito Gonçalves, Juan Ramón Morillo Barragan, Pedro Fuentes Ortega, Antonio Antonio M. Caracol Palomo

**Affiliations:** a Universidad de Extremadura, Centro Universitario de Mérida, Santa Teresa de Jornet 38, 06800 Mérida, Spain; b University of Coimbra, Institute for Systems Engineering and Computers at Coimbra, Department of Mathematics, 3030-290, Coimbra, Portugal; c Universidad de Extremadura, Escuela de Ingenierías Agrarias, Carretera de Cáceres S/N, 06007, Badajoz, Spain; d Escuela de Tráfico de la Guardia Civil, DIRAT, Mérida, Spain; e Cuerpo Nacional de Policía, Mérida, Spain

**Keywords:** Traffic accident, Unmanned aerial vehicle (UAV), Ground control points (GCPs), 3D point cloud, Accuracy assessment

## Abstract

Investigations into traffic accidents that lead to the determination of their causes and consequences are useful to all interested parties, both in the public and private sectors. One of the phases of investigation is the capture of data enabling the complete reconstruction of the accident scene, which is usually the point at which a conflict arises between the slow process of information gathering and the need to restore normal traffic flow. To reduce to a minimum the time the traffic is halted, this paper follows a methodology to reconstruct traffic accidents and puts forward a series of procedures and tools that are applicable to both large and small scenarios. The methodology uses low-cost UAV-SfM in combination with UAS aerial image capture systems and inexpensive GNSS equipment costing less than €900. This paper describes numerous tests and assessments that were carried out on four potential work scenarios (E−1 and E−2 urban roads with several intersections; E−3, an urban crossing with medium slopes; and E−4, a complex road section with different land morphologies), assessing the impact of using simple or double strip flights and the number of GCPs, their spacing distance and different distribution patterns. From the different configurations tested, the best results were achieved in those offset-type distributions where the GCPs were placed on both sides of the working area and at each end, with a spacing between 100 and 50 m and using double strip flights. Our conclusion is that the application of this protocol would be highly efficient and economical in the reconstruction of traffic accidents, provide simplicity in implementation, speed of capture and data processing, and provide reliable results quite economically and with a high degree of accuracy with RMSE values below 5 cm.

## 1 Introduction

In 2022, approximately 20,600 people lost their lives on the roads of the European Union, an increase of 3 % from the previous year [1]. In addition to the human pain and suffering, the economic impact of such deaths is also considerable. The general aim of the Framework of the European Union's policy on road safety [2] is to reduce the number of deaths in road accidents in Europe by half, and this is echoed in the Strategic Action Plan on Road Safety of the European Commission [3].

It is essential to know the causes of road accidents to advance in accident prevention and reduce the number of deaths on the roads.

In general, the police investigation in cases of traffic accidents is carried out in two clearly differentiated stages. The first consists of collecting and investigating the data at the scene of the accident, which then proceeds to questioning the people involved in the event as well as any eye witnesses.

The collection of this information is usually carried out by means of traditional manual procedures, using measurements by tapes or wheels to make sketches or photographs for the task of documentation. This technically-outdated process greatly slows down the collection of the data necessary to the reconstruction of the accident scene, giving rise to the possibility of road delays, closures or diversions, which may, in turn, lead to further accidents.

Consequently, errors are made in the first stage both in the collection of information and in the measurements taken due to the pressure to normalise traffic circulation as quickly as possible The second stage is the reconstruction of the scene by expert personnel who analyse the possible causes that contributed to the accident (road, driver, vehicle, weather conditions, etc.) based on the information collected at the accident scene.

The aim of the reconstruction of traffic accidents is to recreate the event, which is necessary for the analysis of factors such as collision dynamics, collision with pedestrians and cyclists and turning off sloping roads. This type of analysis is needed to produce evidence in the judicial processes following these types of accidents [4].

### 1.1 Literature review

To improve traffic accident investigations, the challenge of the agents in charge of these tasks has long been reliable and timely data collection, and in recent years, possibilities have become enormously more open, thanks to the availability of various technological tools, including total stations, laser scanners, Global Navigation Satellite System receivers (GNSS) and Unmanned Aircraft Systems (UAS). The use of these methodologies and tools provides a series of advantages and disadvantages as described in Topolšek et al. [4]. According to these authors, some of these methods affect the number of researchers required, portability, measurement range, applicability according to the amount of light and climatic conditions, the possibility of remote measurement, data collection duration, scope, the option of posterior data processing, the data collected and, above all, data accuracy. The latter is crucial to establishing the guilt or innocence in court of the participants in a traffic accident since inaccurate data can lead to unfair outcomes.

The work carried out by Osman [5] shows that the use of traditional measurement systems (tape measurement, roll wheel, etc.) can prolong the time that elapses from the accident itself to the conclusion of the measuring process by 2 h or even more.

Similarly Desai et al. [6] reflected that depending on the magnitude of the accident, the time needed to collect effective measurements with these materials can range from 45 min to several hours. Similarly, Struble [7] noted that data collection using traditional techniques can slow traffic flow for hours as the accident scene is reconstructed.

The classical method of tape measurement, which is still the most widely used in the field, also has advantages and disadvantages [4]. The former include its low cost and the fact that no additional equipment or special training is required, while the latter are slower data collection, difficult data processing and poorer quality of data measurement.

The use of new methods for the documentation of traffic accident scenes makes it possible to achieve significant time savings as well as more precise and detailed three-dimensional reconstructions as compared to traditional procedures. The total station is nothing more than a theodolite into which a distance meter has been incorporated. It measures angles and distances, which is information that it processes to obtain spatial coordinates. Distances can be measured using a prism or in a reflectorless mode, allowing just one person to perform the data collection. Electronic total stations allow data capture times to be reduced by up to 33 % [8,9] compared to traditional methods by generating three-dimensional models of an accident with greater detail and accuracy [9].

In a study carried out in the Czech Republic [10], the use of traditional measurement systems was compared with that of total stations, and it was found that the average time saved by using total stations was 49 %. The study also found that conventional measurement tools (wheel, tape, etc.) were not only inaccurate but inappropriate for our time as well as deficient in the accuracy they achieved, particularly when accidents involved sloping roads from which a vehicle was turning off.

Jacobson et al. [8] stated that this type of instrument can collect twice as many measurements in half the time compared to classical methodologies, and it can achieve high accuracy in data capture for graphic scale representation of the accident scene.

Total stations have proven to be very useful in the reconstruction of large traffic accident scenarios [11], providing results with a high degree of accuracy and speed in data acquisition.

The same could be said of GNSS measurement devices as is said of total stations. GNSS is the alternative system. These receivers are commonly used in such fields as topography, geodesy and geomatics, and they can process signals in space that are transmitted by satellites and obtain the geographical position with absolute positional accuracy.

Another technology used in data acquisition from the scene of an accident is the Terrestrial Laser Scanner (TLS), which facilitates the three-dimensional (3D) documentation of accident events in a safer, more flexible, quicker and more accurate way [12]. TLS makes it possible to record in great detail, relative accuracy and at great speed the massive 3D point clouds of the objects located around them. One of the advantages of these devices is that they allow pictures of the scene to be taken while scanning, resulting in images superimposed on the corresponding point clouds to show a natural setting. Another is that the instrument can work under the full range of light conditions, from complete darkness to direct sunlight. From these point clouds, a Digital Surface Model (DSM) can be generated, and 3D animations of the scenes or 3D physical models can be recreated to help analyse what happened in the incident. Laser data acquisition from a scene involves capture from individual scanning stations placed at different locations.

Forman et al. [13] described how each scan requires an average of five to 7 min (configuration and data collection); depending on the extent of the area to be measured, a scene can be taken within around 20 min from the arrival of the equipment. According to this research, the use of these tools has many benefits, such as high speed and detail in measurement, a high degree of conservation of the scene of the incident, measurement without interference with the work area, preview of the scene as a presentation of raw 3D data and visual and quantitative analysis of the characteristics of the area.

The use of TLS for data capture in areas of highway intersections facilitates the quick and reliable recording of data from such infrastructures [12], allowing for shorter outage periods and a permanent 3D record of the area of both highway and accident.

In a comparison of classical data collection with a tape measurement and data collection using a laser scanner, an accident on a curve involving two vehicles and a motorcycle was simulated [14], and the laser capture techniques were quicker at data acquisition, had higher accuracy and were more reliable. In addition, they could be checked and measured long after the accident and used in most situations.

In Slovenia, tests were carried out using different scanners on a simulated accident scenario [15]. Time savings of 77 %, 44 % and 97.3 % were achieved, depending on the type of scanner used. The accuracies were also very high, at between 4 and 6 mm. As in other studies, the tests indicated that the advantage of capturing point clouds makes it possible to carry out subsequent analyses and measurements when the scene of an accident has already been cleared.

One of the disadvantages of TLS is the difficulty in capturing distant objects and the inability to image reflective surfaces [16]. In addition, there are difficulties with scan registration in areas of dense vegetation or very repetitive environments, such as long roads. Although there are TLS capable of capturing objects at great distances, the accuracy may be lower than that of objects that are closer. In a forensic context, objects and work areas should not be more than 60 m from the TLS [11]. Thus, large work scenarios require multiple scans to attempt to address this difficulty, though this increases the accumulation of errors.

Photogrammetry is another of the disciplines used in the reconstruction of accident scenes. It allows the representation and measurement of 3D objects using the information contained in a two-dimensional (2D) image without the need to come into contact with the objects. The photogrammetric process consists of taking several superimposed photographs of the same object or work area, which are later combined to generate a 3D model [17].

With the development of digital cameras and computer hardware and software, photogrammetry has been used in numerous works.

Fraser et al. [18] described a methodology that improves the applicability of near-object photogrammetry in combination with consumer digital cameras for traffic accident reconstruction, decreasing capture times in data collection, which leads to reduced traffic delays.

The use of this methodology allows operating costs to be reduced since only one agent is needed to collect all the data [5], minimizing the capture time of the accident scene with centimetre accuracies.

Digital photogrammetry methods based on Structure from Motion and Multi-View Stereo (SfM-MVS) processes have become efficient image-based methods that allow real-world scenes to be recreated [19].

The incorporation of images captured by Unmanned Aerial Vehicle (UAV) systems in combination with improvements in data processing using algorithms developed from artificial vision and photogrammetry (SfM-MVS photogrammetry, hereinafter UAV-SfM), which are implemented in easy-to-use software tools, are a great step forward in the reconstruction of 3D models.

The use of UAVs has become a powerful and highly profitable tool for capturing images in photogrammetric analysis [20,21] since it is capable of capturing overhead images from different angles and altitudes, thereby monitoring the site of the accident without interrupting the flow of traffic. UAVs also have errors that are well below the generally accepted ranges for the reconstruction of this type of work [22].

As with TLS, the results obtained from the work scenarios once the information has been processed include 3D point clouds, Digital Surface Models (DSMs), orthophotos and orthomosaics.

The use of this type of image taken from UAVs combined with the different photogrammetry–artificial vision algorithms allow data collection time to be shortened by 84 % compared with traditional methods [23]. This approach does not require traffic to be stopped during data collection, and the results obtained are quite accurate.

Some studies have evaluated the quality of the reconstruction of accident scenes in 2D and 3D [24]. These indicate that compared with traditional methods, the use of this type of tool has the advantages of mobility, wide vision and low cost.

This type of vehicle has also been used on simulated scenarios of traffic accidents under extreme environmental conditions [25], in which significant time savings in data collection were achieved along with high accuracy in scene reconstruction compared with manual measurements. This has led to the generalisation of the photogrammetry method based on UAS for traffic accidents in extreme environments.

Some research papers have compared the use of UAVs with GNSS and TLS receivers in accident reconstruction, particularly in the evaluation of the accuracy of the 3D models generated.

In a study carried out by Desai et al. [26], who compared RTK measurements using a GNSS receiver with those obtained from UAV-based mapping, the scale errors derived from the UAVs varied by three cm with respect to the shots taken using a GNSS receiver, which was the accepted threshold for use in public safety cases. This work demonstrates that the cartography obtained by UAVs is a viable option due to its accuracy, affordability and efficiency. Other studies have compared the efficiency and accuracy of TLS and UAV [17], and have found that both the techniques based on TLS and those based on UAS reduced data acquisition time compared to traditional methods and obtained more reliable measurements.

Compared to the TLS-based techniques, UAS-based mapping was much faster, although the errors in measurements were larger, between 1.3 and 2 cm. This paper indicates that one of the drawbacks of automated data collection using TLS and UAV photogrammetry is its lack of sensitivity to details, that is, objects that have not been represented in the 3D model due to their small size. Therefore, in addition to scene measurements, it could also be useful to verify visual details later.

Berezowski et al. [16] indicated that the use of geomatic technologies can be more expensive, which would be a problem for work teams with a reduced budget. The authors concluded that while UAS-based photogrammetry is more suitable for large scenes, TLS is better for smaller ones.

Similar conclusions were reached in another study [27], in which it was found that TLS instruments were more expensive when compared to the use of UAVs and photogrammetry, and that the latter were particularly advantageous in large areas with different sparsely distributed objects for which the use of drone photogrammetry saves hours or days compared to TLS measurements.

Crime scene investigations in Arizona [28] compared the differences between UAV point clouds collected from multiple drones at different flight altitudes and a TLS. The conclusion of these authors was that the UAV point clouds were not as precise as those obtained by the TLS as there was a mean square error in the UAV point clouds of 33.2 mm compared to 2.6 mm for the cloud from the TLS. Nevertheless, the authors indicated that UAV point clouds are accurate enough for forensic analysis at crime scenes or vehicle accident reconstruction.

In the reconstruction of simulations of various scenarios conducted at different flight altitudes with UAVs, the models generated were compared to those acquired using TLS [29]. The authors stated that the accuracy of UAV photogrammetry can be statistically compared with the result obtained by laser scanning. Also reflected in this work is the fact that TLS tools are more expensive than UAS equipment and that less time is required to capture data with the latter, making UAVs a useful and efficient tool that provides sufficient accuracy for most scenarios.

The authors of another research study conducted in Thailand [30] reached the same conclusions. They indicated that the use of TLS is more expensive than the approach proposed of the use of UAVs and photogrammetry.

### 1.2 Objectives and hypotheses

In this article, we present a set of procedures and tools based on a methodology that has as its purpose the reconstruction of different traffic accidents, applicable to large or small scenarios, through the use of low-cost UAV-SfM in combination with data capture systems, using these UAS aerial images and cheap GNSS equipment with prices of less than €900, since they allow the accident reconstruction teams of the Guardia Civil or the Policia Nacional to quickly and efficiently carry out the tasks of capturing, measuring, processing and generating 3D models without excessively interfering in the area of the incident, in an absolute position and with sufficient accuracy to generate a complete 3D reconstruction of the work area.

The proposal is to capture photographs using small drones with a weight not exceeding 250 g because European drone regulations [31] are more permissive with this type of UAV, which is labelled class C0 [32] and allows flights to be carried out in operations of the open category. In this operational category, flight is permitted anywhere, including over people but not over concentrations of people. Another advantage is that with this type of drone, no special training is required in Spain, and the pilots will only be required to familiarise themselves with the manufacturer's user manual.

Among the characteristics to highlight in this type of UAV are its low weight and its ease of transportation and handling, plus the integration of a small camera with a sensor of at least 12 MP, its robustness, ergonomic control knob, transmission systems that allow great connection flexibility in the face of interference, flight autonomy of up to 20–25 min, the possibility of conducting manually or automatically controlled flights, great stability during flights and market prices of less than €500.

Combined with these small UAVs, a Real-Time Kinematic (RTK) GNSS device was used. This GNSS receiver, called rtkREP by the developers, costs less than €400 and allows indirect georeferencing of the images captured by the UAV using the data collected on the Ground Control Point (GCP) in order to intervene in the photogrammetric adjustment of the block of photographs (BA). The advantage of this is to obtain the photogrammetric products (point clouds, orthophotos, etc.) in reference to coordinate systems.

In this context, the reconstruction of several simulated accident scenarios using low-cost UAS and GNSS equipment was undertaken to evaluate the quality of the different products obtained by UAV-SfM. For this task, the positional accuracies of the different simulations were studied using the root mean square error (RMSE) as an estimate of the accuracy between the products generated and the mediated coordinates of a large and dense collection of control points distributed throughout the work area.

## 2 Study areas, equipment and methodology

### 2.1 Study area

**Fig. 1:**
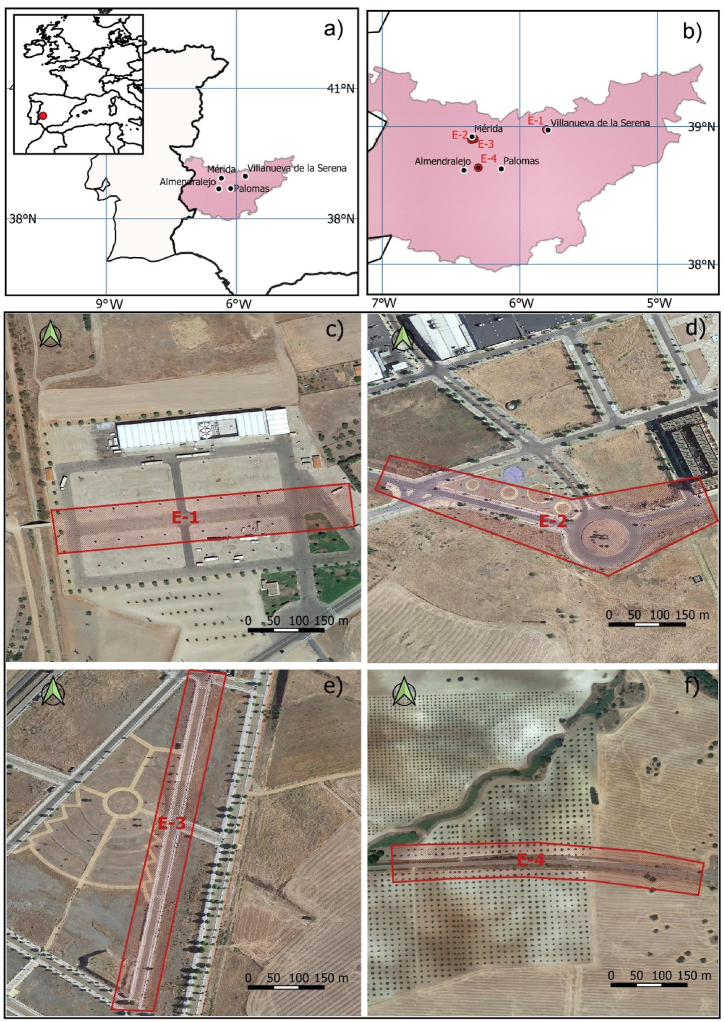
Study areas. (a) Location of the study sites in Spain; (b) location of the study scenarios in the province of Badajoz (Extremadura); (c, d, e, f) google satellite image of each of the scenarios called E−1, E−2, E−3 and E−4.

Due to the complexity of Spanish law regarding flights carried out with UAS over actual road accidents, different sites for the simulation of accident scenes were considered for this work. Four sites with differing characteristics were chosen for the simulations, all within the Autonomous Community of Extremadura (Fig. 1a and b), province of Badajoz (Spain).

The first site, referenced as E−1 (Fig. 1b and c), is in the Villanueva de Serena fairgrounds. The road is of an urban type with several intersections to the left and right. The road has a total width of 13 m and is equipped on both sides for perpendicular parking. It is approximately 220 m long with a practically flat surface. This area was chosen at the request of the local police of Villanueva de Serena to conduct the tests for the simulation of reconstructed traffic accident scenarios since traffic at this site is intense and continuous at certain times.

The second site, E−2 (Fig. 1b–d), is an urban public road in the southwest of the city of Mérida. This road has a width of 12 m for vehicles, of which 6 m are for vehicle circulation and 3 m on each side for in-line parking. The pavement on both sides varies in width between 3 and 6 m. The total length of the road is 360 m with an end-to-end descent of 1 m, intersections and a roundabout of 80 m in diameter.

The third site studied, E−3, is also in Mérida (Fig. 1b–e). It is an urban crossing of about 470 m in length with an end-to-end descent of 8 m resulting from a slope that varies between 1 % and 3 %. The road has a width of 6 m and an area 2.5 m wide on each side for in-line parking and a 2.2 m pavement.

The last of the selected sites, E−4, is a 660 m section of the EX-212 inter-regional highway from Almendralejo to Palomas (Fig. 1b–f) located between PK 9 and 10. It has an 8 m platform with two lanes of 3.5 m and two verges of 0.5 m. It was selected as a complex scenario since it is made up of successive additions of roads, different land morphologies, slopes in clearing and embankment of up to 7 m and abrupt grade changes with curve and slopes of up to 7 %.

### 2.2 Equipment

#### 2.2.1 GNSS receiver

GNSS system technology has been extensively used for different positioning and navigation purposes, and it provides the representation of the results in an absolute reference system. When greater accuracy is required, GNSS geodetic instruments are used in various applications, as they can achieve positioning accuracies of centimetres in real time [33]. Prior to this, only expensive high-quality GNSS equipment has been able to achieve these accuracies. Low-cost availability has now made GNSS instruments a suitable alternative [34,35].

**Fig. 2:**
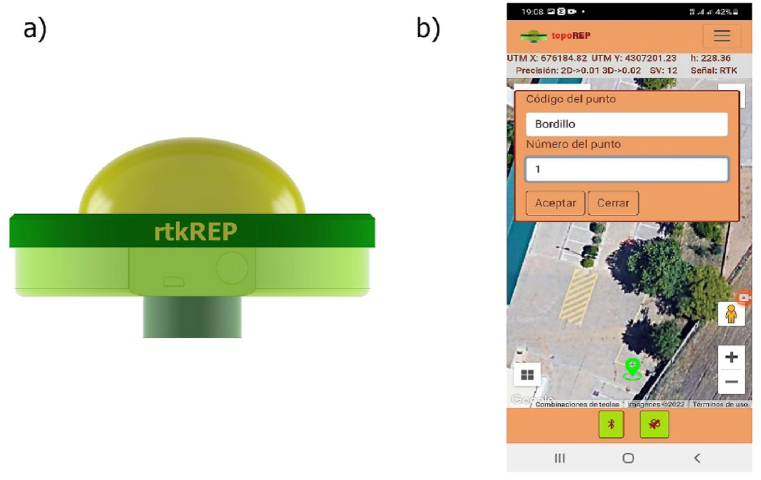
Low cost GNSS receiver, (a) rtkREP device; (b) Interface of the topoREP application in point survey mode.

For this study, RTK GNSS receivers known as rtkREP [36] were used to obtain precise georeferenced positions. This equipment was developed and verified at the School of Agricultural Engineering of the University of Extremadura and consists of an RTK rover, rtkREP (Fig. 2-a) and a topoREP application (Fig. 2-b) capable of connecting to any service provider (NRTK). To do so, device control and Internet connectivity occur using a personal smartphone, while the hardware is protected inside a casing designed and printed in 3D.

This equipment is included within the category of do-it-yourself (DIY) projects, and it has the advantage that it produces significant labour savings as well as an approximation of this type of receiver for users, making 3D files of the pieces available, along with an assembly manual and the relationship of components and software for its control [37,38]. The core of the hardware is based on the simple RTK2B [39] board with dual frequency u-blox ZEDF9P [40] chip and ANN-MB-00 patch antenna.

The final result is a low-cost RTK rover that meets the expectations of users who have been modelling its functions. This is confirmed by the tests conducted to verify its operating parameters: for short and medium baselines, its accuracy and time-to-first fix (TTFF) are at the same level or even better than other geodetic grade receivers [36].

#### 2.2.2 UAS device

One of the main criteria for the evaluation of the type of UAS to use in these studies was compliance with the requirements established in the European UAS regulations for the open category for unmanned aircraft systems with class C0 marking [32] and a weight or maximum take-off weight (MTOW) of less than 250 g. Class C0 drones are allowed in Open Category (A1) [32] operations, an operational category that allows them to fly anywhere as long as there are no location-specific restrictions. They may be flown over people but not over crowds. They must not exceed 19 m/s (68 km/h), and no official training is required.

As this type of UAS has to be implemented and used to practical effect in the reconstruction of traffic accidents by police forces, these drones must also meet a series of conditions, including their small and compact size, ease of handling and transport, high operational range, great stability during flight, sufficient autonomy to fly over the work scene entirely on a single battery charge, equipped with a stabiliser or gimbal that allows the capture of high-resolution images and videos, positioning and guidance with GNSS, and lastly, they must be a good value for the money since there are many manufacturers on the market that meet high expectations in this last criterion.

In our case, we opted for the DJI Mavic Mini 2, the price of which starts at €460. It includes a combo with additional batteries, connections, and so on, and it provides exceptional performance for such a small size with an outstanding camera and a multitude of automatic flight features. Among the most important characteristics are its weight of 249 g and dimensions of 245 × 289 × 56 mm. It has a triple-axis stabiliser incorporating an FC7303 model camera with a 1/2.3″ CMOS sensor and 12 MP size. This sensor allows photographs to be stored in JPEG or RAW format, and it makes videos of up to 4 K with a limit of 30 frames per second. The camera has timer-shooting capability and can capture three different images at different exposures to be combined in an HDR. It has a GNSS module (GPS, Glonass and Galileo) autonomy of up to 31 min (according to the manufacturer) and can fly in winds of up to 38.5 km/h.

Another reason for choosing this equipment was that it can carry out pre-programmed autonomous flights, which means that the flight routes can be optimised such that the photographic shots taken meet the conditions laid down in the flight plan, ensuring that the overlaps between photographs are as uniform as possible, the mission is accomplished and that different angles of capture in the camera are permitted in order to define different trajectories.

### 2.3 Methodology

Having defined and selected the equipment, a work methodology was established with the aim of digitally reconstructing and recording traffic accidents to modify or replace traditional procedures.

As in a classic photogrammetric workflow, the work procedure of this study was structured into two main, distinct and complementary phases, namely, the field work and the office work.

The field work began with the recognition and delimitation of the work area; UAV flight planning (since it had not previously been effectuated in the office); the definition, materialisation and measurement of the coordinates of the GCPs and check points (CPs) using the GNSS receiver; and the execution of the flight in order to obtain aerial photographs of the work area.

The office work included the processing of the data obtained using the GNSS receiver and UAV-SfM and the evaluation of positional accuracy based on the study of the RMSE of the photogrammetric products obtained (point clouds, DSM, orthophotos).

#### 2.3.1 Flight planning

In any photogrammetric work, flight planning comes first, with the aim of providing photographic coverage of the area under study. These autonomous flight schedules allow the UAV to acquire the photographs according to previously defined specifications, such as flight height, speed, overlaps and capture angles, and to determine an optimal trajectory to achieve the correct photographic coverage in the shortest possible time.

One of the hypotheses that was considered was that it is not always possible to plan the flight in the office prior to going out into the field since different circumstances may arise that prevent it, such as not knowing with precision the location of the work area or the need to attend to an unexpected difficulty of some kind. Faced with this possibility, what we proposed was ‘in situ’ autonomous flight planning, or manual planning, in the case of the occurrence of some kind of incident that would prevent the flights from being automatically implemented.

Flight planning is one of the most important phases of the entire photogrammetric process since success will, above all, depend on the quality and geometry of the photographs.

In this work, errors that can appear and be mitigated were considered as long as an adequate capture of the images was achieved according to a certain geometry. DSMs derived from UAV-SfM processing frequently show characteristic systematic errors that affect the vertical component, called ‘doming’ or ‘bowling’. Although the exact origin of these systematic errors is unknown, it seems clear that they are related to a lack of accuracy in the geometric calibration process of the cameras [41]. Most of the UAS use small aerial cameras with low geometric stability that are not designed for photogrammetric purposes, so the internal parameters may suffer variations when turning them on, during handling, and so on. Therefore, pre-calibration of the camera is not useful. Self-calibration is the preferred feature in these cases.

For this study, the internal orientation parameters of the camera were determined by self-calibration, as described below in the data processing section. To improve self-calibration, a set of convergent (non-nadiral) images must be integrated into the adjustment model [42].

Traditionally, photogrammetry flight designs have been almost vertical, with the aim of reducing shadows and improving homogeneity in scale and coverage efficiency. This type of flight, with almost vertical images, should be avoided [43] when using a UAV-SfM fit, since it produces larger doming errors, thus giving rise to poorer overall results. The incorporation of oblique or convergent images into the UAV-SfM workflow has been shown to reduce systematic errors within the resulting models [44].

Studies have been completed in which the uses of oblique images in double-grid flight missions guaranteed a good level of accuracy [45]. Nevertheless, gridded missions consume more flight time and are difficult to execute, especially in scenarios with long linear features. These same authors have carried out tests with hybrid flights that included nadiral and oblique images, obtaining good results in the vertical accuracy of the model. Regarding the camera tilt angle for converging imaging, there is no consensus or documentation justifying the use of particular oblique camera angles [44]. In this work, the authors show a table of nine scientific publications in which image blocks with different inclination angles (10°, 20°, 25°, 30°, 35°, 45°, 60° and 65°) were used with the UAV-SfM workflow.

The autonomous flights were programmed in the work area in the simplest way possible. Two consistent flight routes were programmed for each of the four selected areas where the simulations were carried out. The simplest way to fly over a work area whose characteristics are long and narrow, as in the case of corridors, is to use simple strips. There are studies that support this option [[46], [47], [48]] since it clearly saves overall flight time, number of images, GCP and data processing. The first of the plans consisted of the definition of the flight on the axis of the track of a simple flight line or strip from a point of departure to another of arrival. The second of the planned routes was to follow double parallel flight lines. For the first study area E−1, two additional manual flights were performed, one on a simple flight line and the other following parallel flight lines. For the definition of the geometry of the photogrammetric block, frontal overlaps of between 80 % and 85 %, and 80 % for the sides, were chosen [47,49,50] at a height of 40 m, a GSD of 1.5 cm being obtained between the centres of two consecutive pixels on the ground. This height was chosen to ensure that the photographs for this flight stopover cover the entire route plus an additional margin for the purposes of safety of approximately 50 m. All the flights were performed in duplicate, some capturing images according to a geometry of nadiral photographs and others using convergent geometry with inclination angles of 10°. Four types of flight plans were finally obtained, Nadiral Single Strip (NSS), Convergent Single Strip (CSS), Nadiral Double Strip (NDS) and Convergent Double Strip (CDS).

Despite the doming effect errors produced by nadiral shots, this type of flight was used for two reasons. First, the photographs acquired with this geometry present uniform scales and make it possible to locate details from this perspective that could be used in accident reconstruction. Second, the incorporation of GCP to the BA allows errors produced by doming to be reduced [51].

The application of the DJI Mavic Mini 2 UAS does not provide the option of incorporating autonomous flights by programming waypoints, but after the release by DJI of the SDK libraries, there are currently several applications that allow such programming, including Rainbow for DJI Drones, RedWaypoint, Waypoint OffLine, Litchi, Dronelink, MavenPilot and Map Pilot Pro. Some of these applications do not allow offline flight scheduling and, therefore, require a network connection. Among the mentioned apps, we use RedWaypoint because it allows us to work in offline mode and it has a free version that although limited to a maximum number of waypoints, can feasibly programme flights in a simple way, such as a simple flight line or parallel lines. Most of the applications mentioned have prices ranging from €18 to €100, depending on their features and options.

**Fig. 3:**
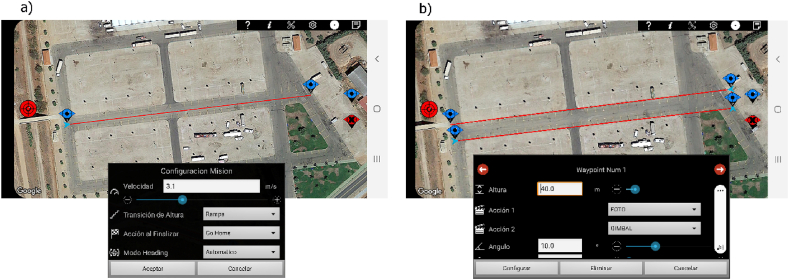
Planning with RedWaypoint in Zone E−1, (a) flight plan with simple flight line and configuration of the speed of the UAV, (b) flight with parallel lines and definition of flight height and actions on waypoint.

Flight scheduling with the app RedWaypoint (Fig. 3a and b) was very simple. Only the waypoints that defined the flight path of the UAV had to be marked, together with the height, speed and type of action to carry out on each of them. Table 2 shows the waypoint number used for each scenario and each flight plan.

In our case, a height of 40 m was defined for the scheduled routes, UAV speed at 3.1 m/s (11.2 km/h) and image capture at 2 s intervals to guarantee the required overlaps. The Mavic Mini 2 drone camera allows shots to be taken at intervals between photos every 2/3/5/10/15/20/30/60s, which allows a wide range of capture options with different overlaps.

We provide a summary of the photogrammetric expressions that allow the distance between photographs *B* (Eq. (1)) to be determined, depending on the frontal overlap *p*, the distance between strips *A* (Eq. (2)) as a function of the lateral overlap *q* and the speed of the UAV, *V*_*UAV*_ (Eq. (3)).

(1) $$B\left(m\right)=\frac{S_{w}\left(mm\right)H\left(m\right)\left(1-p*0.01\right)}{f\left(mm\right)}$$

(2) $$A\left(m\right)=\frac{S_{H}\left(mm\right)H\left(m\right)\left(1-q*0.01\right)}{f\left(mm\right)}$$

(3) $$V_{UAV}\left(m/s\right)=\frac{S_{w}\left(mm\right)H\left(m\right)\left(1-p*0.01\right)}{f\left(mm\right)\Delta t\left(s\right)}$$

where *f* is the focal length (mm); *S*_*H*_ is the sensor height of the camera (mm); *S*_*W*_ is the sensor width of the camera (mm); *p* is the frontal overlap (%); *q* is the side overlap (%); *H* is the flight height (m); and *Dt* is the interval for taking photographs (*s*).

#### 2.3.2 Fieldwork

Once the flight plan had been defined, the next step was to capture the data necessary to provide each photogrammetric block with a number of known spatial position points on the ground for the purpose of adjusting the image blocks for georeferencing with the GCPs and to later use the CPs to carry out the statistical analysis of the final accuracies obtained.

Before performing the planned flights in each of the work areas, the marks that would serve as control points had to be placed on the study areas, thus ensuring their appearance in most of the photographs and guaranteeing an efficient and homogeneous geometric distribution of the support. The general consensus in photogrammetry is to guarantee a uniform distribution of GCPs over the entire study area [51,52].

Lalak et al. [47] conducted an in-depth review of the optimal distribution and number of support points. Regrettably, they concluded that the best arrangement for the GCPs—whether they should be distributed along the edges or in the centre—was unclear, and they failed to determine their optimal arrangement.

In a recent study in which the scope of work was a highway [53], the authors showed that the best results were obtained for those distributions in which GCPs were alternately placed on each side of the road in an offset or zigzagging pattern, with a pair of GCPs placed at each end of the road.

**Fig. 4:**
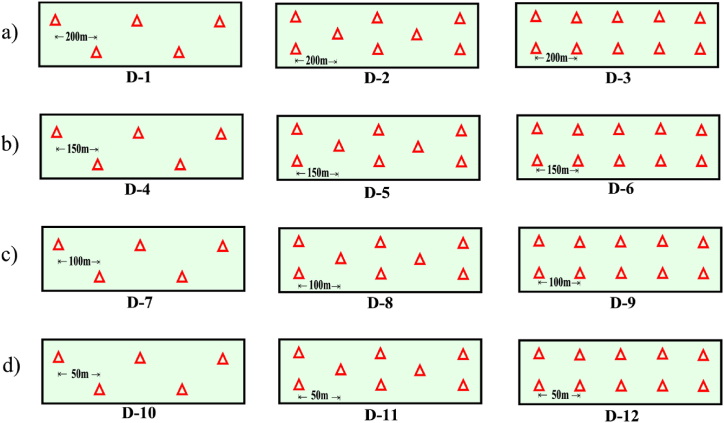
Schemes with the design of the 12 GCP configurations, (a) spacing between the GCPs 200 m and zigzagging pattern (D-1) and offset (D-2, D-3), (b) spacing between the GCPs 150 m and zigzagging pattern (D-4) and offset (D-5, D-6), (c) spacing between the GCPs 100 m and zigzagging pattern (D-7) and offset (D-8, D-9), (d) spacing between the GCPs 50 m and zigzagging pattern (D-10) and offset (D-11, D-12).

In the GCP distribution model described [53], 12 different configurations denominated D-1 to D-12 were designed (Fig. 4). For this purpose, the alternative distribution criteria using an offset and zigzagging pattern were considered, with a separation between the GCPs of 200 (Figs. 4a), 150 (Fig. 4b), 100 (Figs. 4c) and 50 m (Fig. 4d), with the aim of evaluating the influence of the number of GCPs and their distribution on the final accuracies obtained by the different photogrammetric products generated. It was also established that the minimum number of GCPs to be included in one of these configurations was four [[54], [55], [56]], located at the ends to guarantee the adjustment and georeferencing of the block of photographs.

The following study composition was obtained by adapting these twelve possible GCP distribution configurations to each of the four work scenarios (E−1, E−2, E−3, E−4):

E−1: D-3, D-8, D-9, D-10, D-11, D-12.

E−2: D-5, D-6, D-7, D-8, D-9, D-10, D-11, D-12.

E−3: D-2, D-3, D-4, D-5, D-6, D-7, D-8, D-9, D-10, D-11, D-12.

E−4: D-1, D-2, D-3, D-4, D-5, D-6, D-7, D-8, D-9, D-10, D-11, D-12.

Each of these GCP configurations was applied to the four flight plans (NSS, CSS, NDS, CDS), resulting in a total of 148 case studies with scheduled flights.

**Fig. 5:**
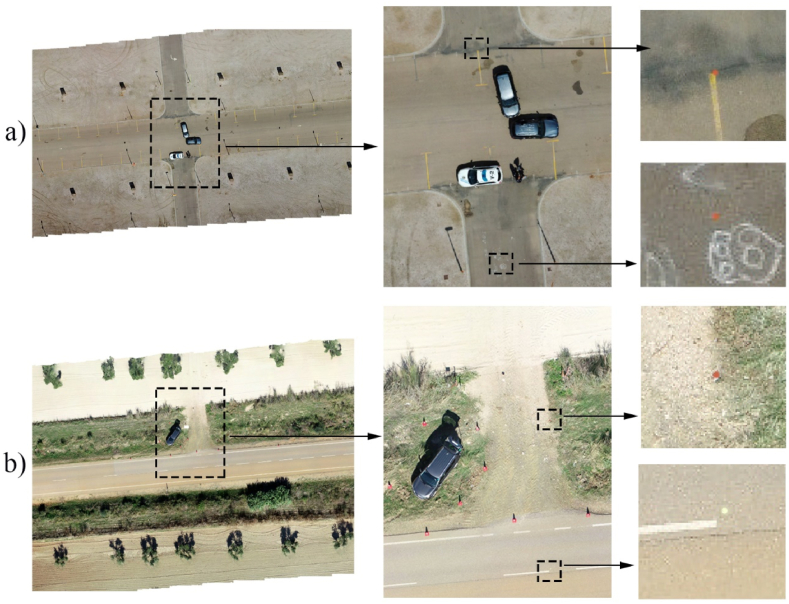
Examples of images showing various GCP and CP targets on portions of orthomosaics of scenarios E−1 (a) and E−4 (b).

Once the GCP design model was defined, marking and measurement were done for each of the scenarios. The GCP and the CP were marked on the ground using paint in assorted colours by means of a circular mark the diameter of which was approximately 5 cm. (Fig. 5a and b).

The GCPs and CPs were measured by conducting a survey with the rtkREP dual-frequency GNSS receiver in network real-time kinematic mode (NRTK), and accuracies between 1 and 2 cm in planimetry and 2–3 cm in elevation [38,52,57] were obtained. Table 1 shows the total number of GCPs and CPs used in each of the distributions established for each scenario.

**Table 1:** Total number of GCPs and CPs used in each of the distributions established for each scenario.

E−1									E−2							
GCP Configuration	NSS		CSS		NDS		CDS		GCP Configuration	NSS		CSS		NDS		CDS
	GCP	CP	GCP	CP	GCP	CP	GCP	CP		GCP	CP	GCP	CP	GCP	CP	GCP
D-3	4	61	4	61	4	61	4	61	D-5	5	109	5	109	5	118	5
D-8	5	60	5	60	5	60	5	60	D-6	6	107	6	108	6	117	6
D-9	6	59	6	59	6	59	6	59	D-7	4	109	4	110	4	119	4
D-10	5	60	5	60	5	60	5	60	D-8	7	107	7	107	7	116	7
D-11	8	57	8	57	8	57	8	57	D-9	8	105	8	106	8	115	8
D-12	10	55	10	55	10	55	10	55	D-10	7	106	7	107	7	116	7
									D-11	11	102	11	103	11	112	11
									D-12	14	100	14	110	14	109	14

E-3									E−4							
GCP Configuration	NSS		CSS		NDS		CDS		GCP Configuration	NSS		CSS		NDS		CDS
	GCP	CP	GCP	CP	GCP	CP	GCP	CP		GCP	CP	GCP	CP	GCP	CP	GCP
D-2	5	145	5	145	5	145	5	145	D-1	4	176	4	176	4	176	4
D-3	6	144	6	144	6	144	6	144	D-2	7	173	7	173	7	173	7
D-4	4	146	4	146	4	146	4	146	D-3	8	172	8	172	8	172	8
D-5	7	143	7	143	7	143	7	143	D-4	5	175	5	175	5	175	5
D-6	8	142	8	142	8	142	8	142	D-5	8	172	8	172	8	172	8
D-7	5	145	14	145	5	145	5	145	D-6	10	170	10	170	10	170	10
D-8	8	142	8	142	8	142	8	142	D-7	7	173	7	173	7	173	7
D-9	10	140	10	140	10	140	10	140	D-8	11	169	11	169	11	169	11
D-10	8	142	8	142	8	142	8	142	D-9	14	166	14	166	14	166	14
D-11	14	136	14	136	14	136	14	136	D-10	13	167	13	167	13	167	13
D-12	18	132	18	132	18	132	18	132	D-11	20	160	20	160	20	160	20
									D-12	26	154	26	154	26	154	26

**Table 2:** Summary of autonomous flights carried out in the work scenarios.

E−1					E−2			
	Waypoint	Time (min)	Distance (m)	Photographs		Waypoint	Time (min)	Distance (m)
NSS	2	1:07	207	37	NSS	3	1:59	366
CSS	2	1:07	207	37	CSS	3	1:59	366
NDS	4	2:23	439	76	NDS	6	4:10	744
CDS	4	2:23	439	76	CDS	6	4:10	744

E-3					E−4			
	Waypoint	Time (min)	Distance (m)	Photographs		Waypoint	Time (min)	Distance (m)
NSS	2	2:23	443	74	NSS	2	3:03	661
CSS	2	2:23	443	74	CSS	2	3:03	6612
NDS	4	4:59	924	150	NDS	4	7:15	1331
CDS	4	4:59	924	151	CDS	4	7:15	1331

**Table 3:** Horizontal and vertical root mean square errors (RMSEs) in the CP (a) and vertical root mean square errors (RMSEs) in CP(DSM) (b) for E−1 scenario configurations and autonomous flights.

(a)									(b)			
Configuration	CSS		NSS		CDS		NDS		CSS	NSS	CDS	NDS
GCP	R_XY_ (cm)	R_Z_ (cm)	R_XY_ (cm)	R_Z_ (cm)	R_XY_ (cm)	R_Z_ (cm)	R_XY_ (cm)	R_Z_ (cm)	R_Z_ (cm)	R_Z_ (cm)	R_Z_ (cm)	R_Z_ (cm)
**D-3**	8.0	40.4	8.4	44.0	7.8	33.5	8.1	35.6	42.2	41.8	37.2	34.9
**D-8**	3.6	4.8	4.0	4.8	3.5	4.6	3.9	4.8	5.5	5.6	5.3	5.2
**D-9**	3.6	4.7	4.0	4.9	3.5	4.7	4.0	5.0	5.2	4.9	5.0	5.1
**D-10**	3.2	5.8	3.6	6.0	3.5	5.3	4.0	5.5	5.4	6.2	5.5	5.7
**D-11**	3.4	4.2	3.9	4.5	3.2	3.7	3.6	4.0	4.9	4.9	4.2	3.8
**D-12**	3.0	4.0	3.4	4.2	3.0	4.2	3.4	3.9	4.8	4.4	4.5	3.6

**Fig. 6:**
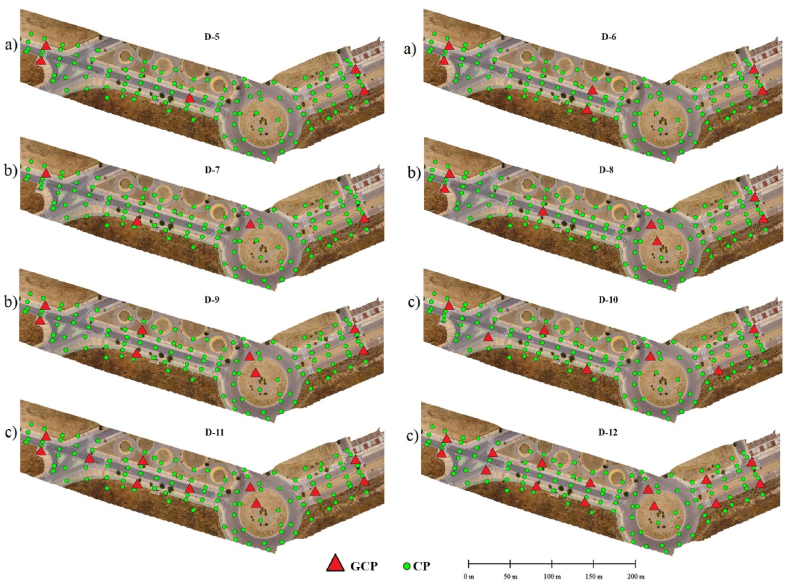
Location of the GCPs (red) according to the distributions established for E−2 and CPs (green) used in this scenario, (a) spacing between the GCPs 150 m and offset pattern (D-5, D-6), (b) spacing between the GCPs 100 m and zigzagging pattern (D-7) and offset (D-8, D-9), (c) spacing between the GCPs 50 m and zigzagging pattern (D-10) and offset (D-11, D-12).

Fig. 6 a, b, c shows the placement of the GCPs for each of the distributions defined for the E−2 scenario as an example.

**Fig. 7:**
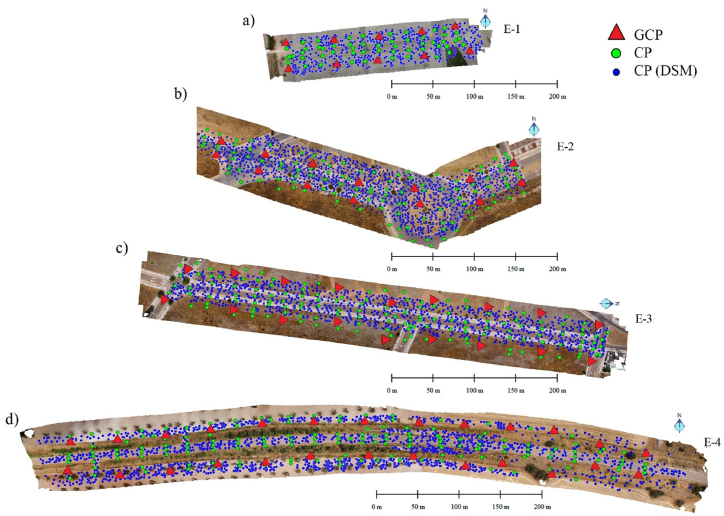
Location on the four scenarios of the GCPs in red, CPs in green and CPs of the Digital Surface Model (DSM) in blue for a spacing between the GCPs 50 m and offset pattern (D-12), (a) E−1, (b) E−2, (c) E−3, (d) E−4.

Finally, to assess the accuracy of the DSM, a GNSS NRTK survey was performed on a set of points randomly distributed over each of the work areas with a higher density than the CP. As a result, a total of 570 captured points were obtained for zone E−1 (Fig. 7a), 1440 for E−2 (Figs. 7b), 1230 for E−3 (Fig. 7c) and a total of 1710 points for E−4 (Fig. 7d). We will call these checkpoints CP (DSM) to differentiate them from the CP materialised with circular marks on each of the scenarios.

Once the measurement work with the GNSS receiver for each scenario was completed, the planned photogrammetric flights were executed. A total of 16 scheduled flights were carried out, four for each of the scenarios and two extra flights performed manually for the first scenario. Table 2 shows the number of waypoints used in the definition of each flight axis, the theoretical flight time, the distance defined by the waypoints and the photographs captured in each flight.

For the manual flights with convergent geometry executed in the E−1 stage, a total of 44 photographs were obtained for the simple strip and 84 for the double strip.

With these manual flights, the number of cases studied was increased to 12, thus bringing the total number of cases treated to 160.

#### 2.3.3 Data processing

For the photogrammetric processing of each of the proposed cases, the Agisoft Metashape Professional 1.7.4 software was used, which is based on the acquisition of large point densities from correlation. This type of software presents an intuitive interface, a fast-learning curve and a diversity of products, allowing the photogrammetric process to be carried out through the processing of digital images and subsequent generation of 3D spatial data. This software was developed under SfM and MVS techniques and procedures in which a 3D model of an object is automatically obtained from multiple convergent images using automatic image-to-image registration methods and image matching algorithms [56].

The workflow is amply described in numerous publications [48,54,56,58] and basically consists of searching for points that are homologous in all images, performing relative orientation and, subsequently, absolute orientation with known position points, GCP. Once the position and orientation of the cameras in space and their internal parameters have been defined, the dense point cloud is captured using MVS and classified. Then a 3D-textured mesh, the digital surface model and the georeferenced orthomosaic are generated.

As a summary, we describe the basic workflow followed in Agisoft Metashape for the generation of the different photogrammetric products for each case.

1. Import the images into Agisoft Metashape along with additional camera metadata (camera calibration, camera orientation angles, camera location accuracy, GPS/INS offset).
2. Process the relative orientation of the images, referred to in this software as image alignment. Detection of characteristic elements (keypoints) in each image occurs in the first of three phases [57]. In the second phase, the correspondence of the keypoints and the elimination of inconsistent correspondences is carried out, and in the third phase, the three-dimensional reconstruction of the scene, the determination of the spatial position of the cameras and the calibration parameters are accomplished with the use of beam adjustment algorithms to finally obtain a dispersed point cloud. (Accuracy: High; key point limit: 40,000; Tie point limit: 4000).
3. Import of GCP coordinates, identification, measurement and definition of the reference system.
4. Optimisation of the model using the calibration parameters of the camera and the geometry of the point cloud. During this process, the block will be adjusted to the selected coordinate system and camera parameters to optimise it and minimise errors. The optimisation procedure includes the reduction of link points acquired in Section 2, the improvement of internal and external orientation parameters and compensation for possible errors caused by the rolling shutter effect [59].
5. Creation of the dense point cloud, which is achieved by applying MVS image matching algorithms. (Quality: low and medium; depth filtering: aggressive).
6. Creation of the DEM from the dense mesh of points.
7. Obtaining the final orthophoto with a resolution of 2 cm.

The models were not optimised in point 4 in order to achieve as simple a workflow as possible and to keep all the elements (trees, signs, vegetation, etc.) that might affect their reconstruction on the surface of the scenarios,. Neither the cleaning of points using the gradual selection tool enabled in the software nor the dense point clouds obtained were subjected to any kind of filtering. The DEM obtained in the processing was therefore, in reality, a DSM.

#### 2.3.4 Positional accuracy assessment

To validate the methodology used in this study, the photogrammetric products obtained were subjected to positional control based on statistics with the aim of determining their accuracy, an essential requirement of all cartographic work. The positional accuracy of a product can be determined by analysing what happens in a significant sample of points or lines [60]. In our case, the CPs previously marked on each of the scenarios were used as well as the points obtained by the GNSS NRTK survey for DSM control.

There are various tests or methods that address the positional quality of cartography and allow the monitoring of positional accuracy X, Y (planimetric or horizontal) and/or Z (altimetric or vertical) of the product. All the tests are based on the comparison of the map or cartographic product with a more accurate source, which in our case, was the data collected on the ground using the GNSS receiver.

The root mean square error (RMSE) is frequently used to assess the geometric accuracy of geospatial products. In our case, we used this method of estimation to assess the quality of the products obtained from the UAV-SfM photogrammetric workflow.

For a two-dimensional variable XY, the RMSE is given by (4):

(4) $$\hat{{RMSE}_{xy}=\sqrt{\frac{1}{n}\sum_{i=0}^{n}\left[{\left(x_{i}-{\hat{x}}_{i}\right)}^{2}+{\left(y_{i}-{\hat{y}}_{i}\right)}^{2}\right]}}$$

where x and $\left({\hat{x}}_{i},{\hat{y}}_{i}\right)\left(x_{i},y_{i}\right)$ are the i-th predicted and measured values, respectively. Extending the last equation to the Z case, the accuracy of the sparse cloud made up of n points can be used and computed as (5):

(5) $$\hat{{RMSE}_{z}=\sqrt{\frac{1}{n}\sum_{i=0}^{n}{\left(z_{i}-{\hat{z}}_{i}\right)}^{2}}}$$

## 3 Results and analysis

Once the photogrammetric blocks had been processed for each of the proposed cases, the DEM (DSM) of each was generated with an average density of 90 points/m2, a resolution of about 10 cm/pixel and an orthophoto with an output resolution of 2 cm/pixel. These products served as the basis for evaluating positional accuracy, studying the differences between each of the three positional components (X, Y, Z) obtained from the comparisons resulting from measuring the coordinates of each of the cartographic products. The GCP and CP, and the coordinates obtained by GNSS receiver.

The following are the results obtained in measurements carried out on the CP, and the CP (DSM) were then distributed over each of the scenarios. From the CP distributed over each of the cases studied, the planimetric and altimetric accuracies were analysed by means of the RMSE_XY_ and RMSE_Z_. The CP (DSM) enables us to evaluate the accuracy of the DSM with greater rigour, since the RMSE_Z_ obtained from these points contains a much larger data sample.

To reduce column widths, the results are displayed for each of the scenarios, referring in each of the tables to RMSE_XY_ as R_XY_, and RMSE_Z_ as R_Z_.

### 3.1 Scenario 1

**Fig. 8:**
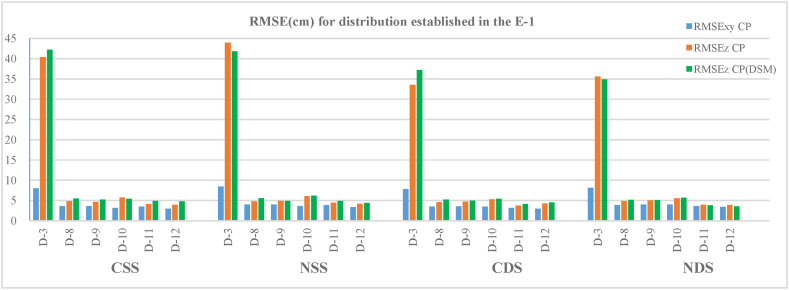
Horizontal and vertical root mean square errors (RMSEs) in the CP and vertical root mean square errors (RMSEs) in CP(DSM) for E−1 scenario configurations and autonomous flights.

Table 3 and Fig. 8 show the RMSE obtained from the CP sample CP (a) and from the CP (DSM) (b) used to define the evaluation of the accuracy of the first scenario (E−1) of autonomous flights. The study area has a length of 220 m, and 55 to 61 CP and 570 CP (DSM) were used to determine the horizontal and vertical RMSE results show that the planimetric component (RMSE_XY_) presents small errors for the configurations studied in this scenario. The distribution that shows the greatest error in planimetry is D-3 with values of around 8 cm both for flights with converging and nadiral shots and for single and double strips. The remaining configurations (D-8, D-9, D-10, D-11 and D12) present lower errors, with values of between 3 and 4 cm. It is already evident from the few results analysed that the increase in the number of GCPs from 4 (D-3) to 6 or more GCPs produces an improvement in the results of the horizontal component, which in some cases, is more than double that of the D-3 configuration. The best planimetric accuracy is achieved with 10 GCP in the D-12 configuration. If we compare this last configuration with D-9, both with the same distribution of GCPs but with a different amount (D-9 with 6 GCPs), the differences in error are less than 0.5 cm.

Regarding the altimetric component, the results obtained for both CP and CP(DSM) are quite similar for the six configurations of the samples that were studied. The largest errors are also present in the D-3 configuration. The values of the errors vary in this configuration between 33 and 44 cm for the four types of flights (CSS, NSS, CDS and NDS) and are four or five times higher than those of the planimetric component. The rise from four GCPs to five or more produces falls in the magnitude of the altimetric errors by up to ×8 or ×10 depending on the case and its configuration.

From the analysis of these data we can state that, except for D-3, the configurations present similar results. Single (CSS, NSS) and double (CDS, NDS) strips show similar errors in both planimetry and altimetry. Nevertheless, the single strips show slightly higher values compared to the double strips in some of the configurations, not including D-3.

**Fig. 9:**
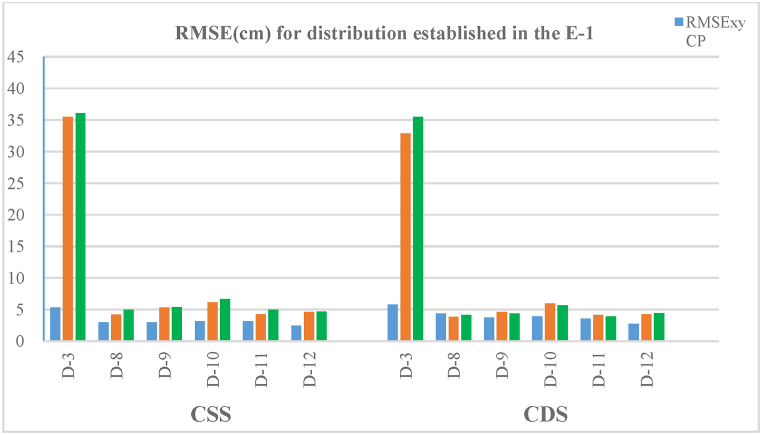
Horizontal and vertical root mean square errors (RMSEs) in the CP and vertical root mean square errors (RMSEs) in CP(DSM) for E−1 scenario configurations and manual flights.

Table 4 and Fig. 9 show the results obtained from the manual flights carried out in the E−1 scenario, with the aim of comparing the results of autonomous flights with manual ones. The results show the same values for each in this scenario.

The greatest errors are again found in the D-3 configuration, which presents altimetric errors of around 35 cm, suggesting that this type of distribution, with only four GCPs located at the ends with a separation of 200 m, is not the most suitable for this type of linear work. Despite the adequacy of the resulting planimetric accuracy, the same does not occur with the altimetric values.

Once more in these tests D-12 is the configuration with the least error. Nevertheless, the difference with respect to the other configurations (D-8 to D-11) is small, 0.7 cm in planimetry and almost 2 cm in altimetry when compared, for example, with D-10.

### 3.2 Scenario 2

**Fig. 10:**
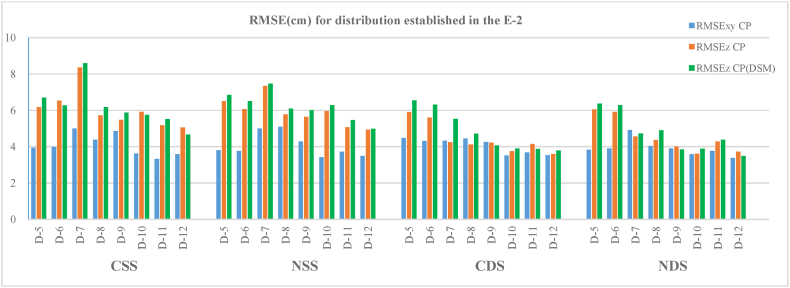
Horizontal and vertical root mean square errors (RMSEs) in the CP and vertical root mean square errors (RMSEs) in CP(DSM) for the E−3 scenario configurations and autonomous flights.

The results obtained for the cases studied for scenario E−2 are shown in Table 5 and Fig. 10. Between 100 and 109 CP and 1440 CP(DSM) were used to evaluate accuracy on this 360 m stretch of road.

**Table 4:** Horizontal and vertical root mean square error (RMSEs) in the CP (**a**) and vertical root mean square error (RMSEs) in CP(DSM) (**b**) for the E−1 scenario configurations and manual flights.

(a)					(b)	
Configuration	CSS		CDS		CSS	NSS
GCP	R_XY_ (cm)	R_Z_ (cm)	R_XY_ (cm)	R_Z_ (cm)	R_Z_ (cm)	R_Z_ (cm)
**D-3**	5.3	35.5	5.8	32.9	36.1	35.5
**D-8**	3.0	4.2	4.4	3.8	5.0	4.1
**D-9**	3.0	5.3	3.7	4.6	5.4	4.4
**D-10**	3.2	6.1	3.9	5.9	6.6	5.7
**D-11**	3.2	4.3	3.6	4.1	5.0	3.9
**D-12**	2.5	4.6	2.8	4.2	4.7	4.4

**Table 5:** Horizontal and vertical root mean square errors (RMSEs) in the CP (**a**) and vertical root mean square errors (RMSEs) in CP(DSM) (**b**) for the E−2 scenario configurations and autonomous flights.

(a)									(b)			
Configuration	CSS		NSS		CDS		NDS		CSS	NSS	CDS	NDS
GCP	R_XY_ (cm)	R_Z_ (cm)	R_XY_ (cm)	R_Z_ (cm)	R_XY_ (cm)	R_Z_ (cm)	R_XY_ (cm)	R_Z_ (cm)	R_Z_ (cm)	R_Z_ (cm)	R_Z_ (cm)	R_Z_ (cm)
**D-5**	3.9	6.2	3.8	6.5	4.5	5.9	3.8	6.1	6.7	6.8	6.5	6.4
**D-6**	4.0	6.5	3.8	6.1	4.3	5.6	3.9	5.9	6.3	6.5	6.3	6.3
**D-7**	5.0	8.4	5.0	7.3	4.3	4.2	4.9	4.6	8.6	7.5	5.5	4.7
**D-8**	4.4	5.7	5.1	5.8	4.4	4.1	4.0	4.4	6.2	6.1	4.7	4.9
**D-9**	4.9	5.5	4.3	5.6	4.3	4.2	3.9	4.0	5.9	6.0	4.1	3.8
**D-10**	3.6	5.9	3.4	6.0	3.5	3.7	3.6	3.6	5.8	6.3	3.9	3.9
**D-11**	3.3	5.2	3.7	5.1	3.7	4.1	3.8	4.3	5.5	5.5	3.9	4.4
**D-12**	3.6	5.0	3.5	4.9	3.5	3.6	3.4	3.7	4.7	5.0	3.8	3.5

Also for this scenario, the RMSE_Z_ obtained for both the CP and the CP(DSM) samples behave in a similar way and show very similar errors.

If we compare the common results of this E−2 scenario with the previous E−1, the GCP configurations D-8 to D-12 present similar trends in planimetric and altimetric errors as the number of GCPs increases in the adjustment for both single and double shots acquired using convergent and nadiral geometry (CSS, NSS, CDS, NDS).

In this scenario, 4 GCPs were used for the D-7 case in the BA. The results show the highest RMSE_Z_ in the single strips (CSS, NSS), with mean values of 8 cm and 5 cm in the planimetric ones. In the double strips, the altimetric errors decreased to mean values of 5 cm and 4.5 cm for the planimetric component. In cases D-5 and D-6, where 5 and 6 GCPs were used, respectively, the altimetric errors decreased by between 6.1 and 6.5 cm in the single strips and between 5.6 and 6.1 cm in the double strips. The planimetric values obtained for these two cases varied between 3.8 and 4.5 cm. In cases D-8 to D-12, in which the number of GCPs used in the adjustment varied between 7 and 14, both planimetric and altimetric errors decreased.

Double strips presented more precise solutions than single strips in most cases, with accuracy improving by an average of 24 %. Nevertheless, all the scenarios will be analysed before attempting to draw conclusions.

Also for this scenario, the D-12 configuration presents a lower RMSE of around 3 and 4 cm for both planimetry and altimetry.

As there are a larger number of cases in this scenario, the results obtained can be analysed based on the interval between the GCPs. Thus, in the 150 m space interval, cases D-5 and D-6 show similar errors for all configurations, both for the planimetric component (3.8–4.5 cm) and the altimetric one (5.6–6.7 cm). When the distance between the GCPs decreases to 100 m, as in cases D-7, D-8 and D-9, the errors are not as homogeneous as there is a clear difference between the results obtained for the first case (D-7) and the other two (D-8 and D-9). It is in D-7, which has a zigzagging GCP distribution, that the greatest errors are found in the single strips (CSS and NSS) with average RMSE_Z_ of between 8.5 and 7.4 cm and an average RMSE_XY_ of around 5.0 cm. For the double strips (CDS and NDS), the planimetric errors decrease to values of between 4.9 and 4.3 cm and altimetric errors of between 4.6 and 4.9 cm for the mean of the CP and CP(DSM). Nevertheless, for the offset GCP configuration (D-8 and D-9), the errors obtained for both single and double strips are lower, with average values of CP and CP(DSM) in the altimetric component for the strips, single strips between 5.7 and 6.0 cm and double strips between 3.9 and 4.6 cm.

The models with the greatest accuracy are found in the last three cases with 50 m spacing between the GCPs because reducing the distance between GCPs produces an increase in the number of GCPs used in the adjustment. In the case of D-10, with a zigzagging distribution and a total of 7 GCPs, the errors in planimetry that were obtained are similar for single and double strips with values of between 3.4 and 3.6 cm, though in this case, the altimetric errors show differences of up to 2 cm between single and double strips. Thus, for the former (CSS and NSS), the mean RMSE_Z_ varies from 5.8 to 6.3 cm, and for the latter, (CDS and NDS), it varies between 3.5 and 3.9 cm. Cases D-11 and D-12 present the lowest errors of all scenarios, with fairly uniform values in the planimetric component for the four strips of between 3.3 and 3.8 cm. The altimetric component presents a greater difference between single and double strips. The mean RMSE_Z_ for single strips is between 4.7 and 5.2 cm, and for double strips, it is between 3.5 and 4.4 cm.

### 3.3 Scenario 3

**Fig. 11:**
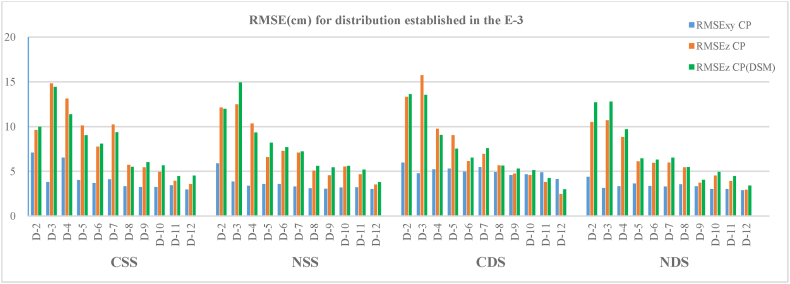
Horizontal and vertical root mean square error (RMSEs) in the CP and vertical root mean square errors (RMSEs) in CP(DSM) for the E−3 scenario configurations and autonomous flights.

In this scenario, the number of cases studied is greater than that of the previous ones since the length of the road was greater. For this scenario, between 132 and 145 PC (Table 1) and 1230 PC(DSM) were used. Table 6 and Fig. 11 show both planimetric and altimetric RMSEs.

**Table 6:** Horizontal and vertical root mean square error (RMSEs) in the CP (**a**) and vertical root mean square error (RMSEs) in CP(DSM) (**b**) for the E−3 scenario configurations and autonomous flights.

(a)									(b)			
Configuration	CSS		NSS		CDS		NDS		CSS	NSS	CDS	NDS
GCP	R_XY_ (cm)	R_Z_ (cm)	R_XY_ (cm)	R_Z_ (cm)	R_XY_ (cm)	R_Z_ (cm)	R_XY_ (cm)	R_Z_ (cm)	R_Z_ (cm)	R_Z_ (cm)	R_Z_ (cm)	R_Z_ (cm)
**D-2**	7.1	9.6	5.9	12.1	6.0	13.3	4.4	10.5	10.0	12.0	13.6	12.7
**D-3**	3.8	14.8	3.9	12.5	4.8	15.7	3.1	10.7	14.4	14.9	13.5	12.8
**D-4**	6.5	13.1	3.4	10.3	5.2	9.7	3.3	8.8	11.4	9.3	9.1	9.7
**D-5**	4.0	10.1	3.6	6.6	5.3	9.0	3.6	6.1	9.0	8.2	7.5	6.4
**D-6**	3.7	7.7	3.6	7.3	5.0	6.1	3.4	5.9	8.1	7.7	6.5	6.3
**D-7**	4.1	10.2	3.3	7.1	5.5	6.9	3.3	6.0	9.4	7.2	7.6	6.5
**D-8**	3.3	5.7	3.1	5.1	4.9	5.7	3.6	5.4	5.5	5.6	5.6	5.5
**D-9**	3.2	5.4	3.1	4.6	4.6	4.8	3.3	3.7	6.0	5.4	5.3	4.0
**D-10**	3.3	4.9	3.2	5.5	4.7	4.6	3.0	4.5	5.7	5.6	5.1	5.0
**D-11**	3.5	3.9	3.2	4.7	4.9	3.8	3.0	3.9	4.5	5.2	4.2	4.5
**D-12**	3.0	3.6	3.0	3.5	4.1	2.5	2.9	2.9	4.5	3.8	3.0	3.4

For all the cases studied, both horizontal and vertical accuracy improve as the number of GCPs used in the adjustment and their location in the work area increase, and planimetric accuracy is always better than altimetric.

For this case, the RMSE_XY_ maintains the trends seen in the previous scenarios, with values varying between 7.1 cm in the D-2 case of the convergent single strip and up to 3.0 cm in the D-12 case in the double strips. The values of these errors tend to decrease and stabilise from the D-5 case on both single and convergent shots with values between 5.3 and 2.9 cm, this case being the point from which the number of GCPs begins to increase.

The results show that for both this scenario and the previous ones, horizontal errors are similar and lesser when the separation between the GCPs is equal to or less than 150 m. A fall is observed in the mean values of these errors of between 0.5 and 1.0 cm with respect to the separations of 100 and 50 m. In case D-4 of the convergent single strip (CSS), a value of RMSE_XY_ of above the average of its 150 m separation environment between the GCPs is observed, which is because in this case, the distribution pattern of the GCPs formed a zigzag, with only four GCPs used in the distribution for the scenario adjustment. This type of zigzagging pattern presents the problem that the distance between GCPs for the most favourable case is 150 m, while the most unfavourable separation is twice that, in this case, 300 m, and this type of arrangement may give rise to lower accuracies depending on the number of GCPs used.

The planimetric accuracies are better than the altimetric ones for most cases, both in this scenario and in the previous ones. Also, in this scenario, the RMSE_Z_ obtained in the CP and in the CP(DSM) are once again practically the same, with average differences of less than 8 %. The most significant differences are found in cases D-2, D-3 and D-4, with variations of between 10 % and 14 % in both single and double strips. The values of these minimal differences confirm that the CP sample used for the evaluation of positional accuracy is appropriate.

An analysis of the results obtained for each of the cases grouped by separation between the GCPs allows us to deduce that:

When GCPs were used with separations of 200 m, as in cases D-2 and D-3, the highest RMSE_Z_ in both single and double, convergent and nadiral strips were presented. For example, for single strips, case D-3 presents errors in CP of 14.8 cm (CSS) and 12.5 cm (NSS), and in double strips, values of 15.7 cm (CDS) and 10.7 cm (NDS). The errors found in the CP(DEM) are 14.4, 14.9, 13.5 and 12.8 cm, respectively, which represents variations of between 4 % and 16 % with respect to the errors of the CP. For these two cases, the number of GCPs used during the BA of photographs was five and six.

For the configuration of cases D-4, D-5 and D-6 with a distribution of 150 m between the GCPs, an improvement was seen in the results obtained with respect to the previous cases. The altimetric errors decreased when using double strips (CDS and NDS) compared to single strips (CSS, NSS) and when using a GCP distribution on both sides of the road with an offset pattern (D-6) instead of a zigzag distribution (D-4). For this separation interval, a total of four GCPs were used in D-4, which is where the highest errors were observed, particularly in the altimetric component, with values of between 10.3 and 13.1 cm in the CPs and 9.3–11.4 cm in the CP(DSM) in the single strips. In the double strips, for this configuration, the errors obtained were 9.7, 8.8, 9.1 and 9.7 cm (CSS, NSS, CDS and NDS). For cases D-5 and D-6, the number of GCPs used was seven and eight, respectively, and the results show a decrease in both planimetric and altimetric errors with respect to case D-4, reaching differences of up to 5.4 cm in single strips and an average error between double strips of 2.9 cm.

The use of the distribution with offset type pattern (D-6) produced an increase in the number of GCPs used in the BA of photographs. Among the two offset-type configurations D-5 and D-6, the latter obtained better altimetric accuracy, probably due to the double symmetrical distribution of the GCPs along each side of the road.

Similar results were found for cases D-7, D-8 and D-9 in which GCPs were separated by 100 m. Table 6 shows that the RMSE_Z_ decreases when using both nadiral and convergent double strips and that these values also decrease when using an offset GCP distribution (D-9) versus a zigzagging distribution (D-7) since this type of distribution tends to decrease the number of control points used in the adjustment. For the D-8 case, which has an offset distribution of GCP and two extreme points alternating with a central one, the RMSE_Z_ presents a loss of altimetric accuracy compared to those obtained in the case of D-9, with a difference of between 1.7 cm and 3.5 cm. For these two cases, eight and 10 GCP were used compared to the five GCP used in case D-7. Cases D-8 and D-9 significantly improve both horizontal and vertical accuracies compared to D-7, with differences between 0.5 and 4.5 cm in altimetry and between 0.2 and 0.9 cm in planimetry.

To conclude the evaluation of the results of this scenario, the data presented in Table 6 and Fig. 11 show that for the distance of 50 m between GCPs—cases D-10, D-11 and D-12—the results were most accurate in altimetry. As in the previous cases studied, a tendency for altimetric errors to decrease when using double strips (CDS, NDS) is also observed, but in these cases, the values compared with those of single strips (CSS, NSS) are relatively small, at less than 1 cm. This similarity in the results obtained for the single and double strips is due to the fact that by using short separation distances between GCPs (50 m), the number of GCPs increases when compared with the rest of the cases with greater spacing (Table 1) and, therefore, the greater number of GCPs used in the BA leads to the reduction in errors. For these cases, the tendency for RMSE_Z_ to decrease remains from the distribution with GCPs zigzagging (D-10) to one of the offset types (D-12). Again, as in previous cases, the results obtained for the offset GCP distribution with double points at each end (D-12) are more precise than those of the offset distribution with alternation of two extreme points and a central one (D-11).

### 3.4 Scenario 4

**Fig. 12:**
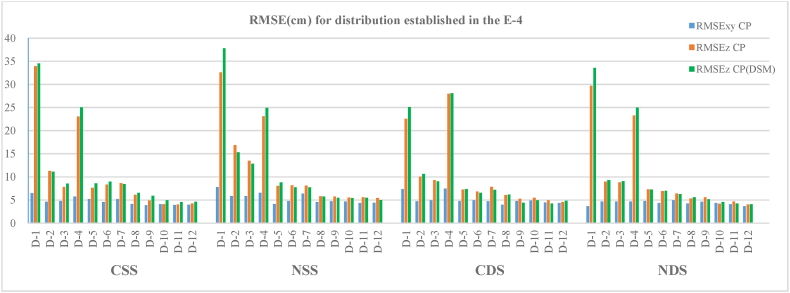
Horizontal and vertical root mean square errors (RMSEs) in the CP and vertical root mean square errors (RMSEs) in CP(DSM) for the E−4 scenario configurations and autonomous flights.

This last scenario is the most complex and the longest of all those studied. To determine the positional accuracies in the different cases proposed in this scenario, the RMSE were calculated for a total of between 154 and 176 CP and 1710 for the CP(DSM). The results are shown in Table 7 and Fig. 12.

**Table 7:** Horizontal and vertical root mean square errors (RMSEs) in the CP (**a**) and vertical root mean square errors (RMSEs) in CP(DSM) (**b**) for the E−4 scenario configurations and autonomous flights.

(a)									(b)			
Configuration	CSS		NSS		CDS		NDS		CSS	NSS	CDS	NDS
GCP	R_XY_ (cm)	R_Z_ (cm)	R_XY_ (cm)	R_Z_ (cm)	R_XY_ (cm)	R_Z_ (cm)	R_XY_ (cm)	R_Z_ (cm)	R_Z_ (cm)	R_Z_ (cm)	R_Z_ (cm)	R_Z_ (cm)
**D-1**	6.5	33.9	7.7	32.6	7.3	22.6	3.7	29.7	34.5	37.8	25.1	33.6
**D-2**	4.6	11.3	5.8	16.9	4.7	10.0	4.7	8.9	11.1	15.3	10.6	9.3
**D-3**	4.8	7.8	5.8	13.5	4.9	9.3	4.7	8.8	8.5	12.8	9.0	9.1
**D-4**	5.7	23.0	6.5	23.1	7.5	28.0	4.7	23.2	25.0	24.9	28.1	25.0
**D-5**	5.2	7.6	4.1	8.1	4.8	7.2	4.8	7.3	8.6	8.8	7.3	7.2
**D-6**	4.6	8.3	4.8	8.2	4.9	6.8	4.4	6.9	9.0	7.7	6.6	7.0
**D-7**	5.2	8.6	6.4	8.1	4.7	7.8	5.0	6.4	8.4	7.7	7.2	6.3
**D-8**	4.1	6.1	4.6	5.8	4.0	6.1	4.2	5.3	6.5	5.7	6.2	5.6
**D-9**	3.9	4.9	4.7	5.7	4.8	5.2	4.6	5.6	5.9	5.5	4.4	5.2
**D-10**	4.1	4.1	4.7	5.5	4.9	5.5	4.3	4.2	4.9	5.4	4.9	4.6
**D-11**	3.9	4.0	4.4	5.6	4.4	4.9	4.1	4.7	4.6	5.5	4.3	4.3
**D-12**	4.0	4.2	4.4	5.5	4.4	4.6	3.7	4.0	4.6	5.0	4.8	4.1

As in the previous cases, both planimetric and altimetric RMSE are observed to decrease when the number of GCPs is expanded in the photogrammetric adjustment of the photographic blocks as is their distribution over the work scene. For this scenario, the planimetric accuracies obtained in the twelve cases studied are also better than the altimetric ones.

A comparison of the results from the single (CSS, NSS) and double strips (CDS, NDS) in each case show that they are practically the same, with slightly fewer errors in the double strips. Nevertheless, when the number of GCPs used is small, such as in the D-2 case (7 GCPs), the double nadiral and convergent strips show fewer errors than the flights made with single strips (CSS, NSS).

The RMSE_XY_ have the same patterns as in the previous examples, with values ranging between 7.7 and 6.5 cm in single strips for the D-1 case and up to 4.4 and 3.7 cm for double strips in the D-12 case.

In this scenario, the differences between the altimetric errors obtained from the CP and the CP(DEM) are also identical, with average variations of 7 %.

Fig. 12 reveals that the highest altimetric errors for both single and double strips are found in cases D-1 and D-4. In these two cases, a total of four and five GCPs were used for the BA of photographs. In the remaining cases, more than six GCPs were used in the adjustment, leading to more precise solutions than for these two cases. The use of a small number of GCPs produces a deterioration in the accuracy of the models.

An analysis of the results according to the distance in the separation between GCPs shows that for the 200 m interval case, D-1 has the highest planimetry errors in both single and double strips, and these errors decrease in cases D-2 and D-3. For D-1, the zigzagging distribution of GCPs in the scenario means that the total number of GCPs used in the adjustment is four, while in the offset-type distribution, the number of GCPs increases to seven for case D-2 and eight for D-3. A rise in the number of GCPs leads to a fall in planimetry errors. The same trend is observed for the altimetric errors as for the planimetric ones. In the case of D-1, quite high errors were obtained, which in some of the flights were up to four times higher than for the D-2 or D-3 cases. The inclusion in the block of a greater number of GCPs, as in cases D-2 and D-3 compared with D-1, drastically reduced errors. For example, for CSS flight and using the CP sample, the RMSE_Z_ was 33.9 cm for D-1, 11.3 cm for D-2 and 7.8 cm for D-3.

The positional accuracies obtained from the data sample of the CP(DEM) reveal RMSE_Z_ of 34.5 cm for D-1, 11.1 cm for D-2 and 8.5 cm for D-3. The results were similar for the remaining flights that were analysed (NSS, CDS, NDS).

Upon examination, the data in Table 7 and Fig. 12 lead to the same conclusion as when the distance between the GCPs was 150 m (cases D-4, D-5 and D-6). For the four flights in this interval, the RMSE_XY_ tend to decrease as the configuration of the GCP distribution changes, and their number increases in each case. Thus, for D-4, four GCPs were used; in D-5, a total of eight were used; and in D-16, a total of 10 were used. Nevertheless, a more dramatic difference in errors among D-4, D-5 and D-6 is observed in altimetry.

Case D-4, in which four GCPs were used for the BA, presents altimetric errors up to three times greater than for cases D-5 and D-6, which once more verifies that a minimum number of points and an inadequate distribution produce erroneous models, and these are particularly noteworthy in the altimetric component.

The 100 m separation interval between the GCPs also presents the previously observed guidelines regarding the distribution and number of GCPs over the work area. For this section, the RMSE_Z_ obtained in the single convergent and nadiral strips present quite similar values, though slightly higher than those reached in the double strips, with differences of between 1.9 and 0.7 cm. The largest planimetric and altimetric errors are presented for the D-7 case in the four flights. The configuration of this case is of the zigzagging type, and a total of seven GCPs were distributed, reaching a higher RMSE than cases D-8 and D-9, which present an offset-type distribution and a greater number of GCPs. The altimetric errors in D-8 are slightly higher than those of D-9, with average maximum differences of 1 cm and minimum differences of 0.2 cm in the four flights that were analysed.

In the last of the sections studied, with a distance of 50 m between the GCPs, we find quite similar results but with the smallest horizontal and vertical errors of the entire scenario dataset. For a zigzagging configuration (D-10) with 13 GCPs used in the adjustment, the RMSE_XY_ and RMSE_Z_ obtained were slightly higher than those of D-11 and D12, with variations of less than 5 %. The extreme values of the RMSE_XY_ varied between 3.7 and 4.9 cm and the average RMSE_Z_ between 4 and 5 cm.

## 4 Discussion

The final quality of the different photogrammetric products derived from the combined UAV and SfM workflow through the use of low-cost tools for the reconstruction of traffic scenarios will largely depend on the strategy chosen in planning the georeferencing of the work area [43,[48], [49], [50],[52], [53], [54],^61^] and, to some degree, on the definition of the flight geometry and the images acquired [47,[49], [50], [51]].

The results obtained in this study confirm that the accuracy of the 3D models that are generated will depend on the amount of GCPs incorporated in the BA of images, their distribution over the work area and the separation distance between them.

As in other studies [48,50,54,62], the evaluation of the geometric accuracy of the 3D models generated in the different cases studied in the four scenarios led to the finding that the introduction of large separation distances between GCPs produces quite large RMSE at the control points, which are particularly relevant in the vertical component [52,54,[63], [64], [65], [66]].

### 4.1 GCP spacing of 200 m

Although some recent studies agree that the final precision of the products obtained (DSM, orthophotos) using UAV-SfM techniques depends largely on the amount of GCP used and the distribution over the study area, there are a few studies that focus on runners [53].

For any of the three scenarios in which a 200 m separation interval between the GCPs was used, a comparison can be made with the similar distance of 180 m used by Carvajal-Ramirez et al. [67] in their study on landslides. The RMSE, especially the altimetric ones, varies by 34–44 cm for cases D-3 (E−1), D-2 and D-3 and by 10–15 cm for scenario E−3 and for the last of the scenarios (E−4). The results for D-1 are between 22 and 37 cm and between 9 and 16 cm in cases D-2 and D-3. The largest errors are obtained at this distance and with a zigzagging distribution (D-1), and these errors are considerably decreased when a GCP distribution is introduced at each end of the work area with an offset pattern. The latter type of distribution with more uniform coverage in GCPs is in line with the results in the literature [47,51,52,68].

The use of each type of distribution entails the distribution of a number of GCPs, so that for a zigzagging distribution, the number of GCPs will always be less than for a distribution with an offset pattern. When the spacing used between GCPs is particularly wide, errors increase [64,68].

For this distance of 200 m, the largest errors were found in D-3 (E−1) and D-1 (E−4), the first of which used an offset-type pattern while the second was zigzag, but as a result of these distributions, the total number of GCPs used was four in both, with results similar to those obtained by Tahar [63] in a work area with different configurations and numbers of GCPs. Nevertheless, for cases D-2 and D-3 (E−3 and E−4), both scenarios with offset type patterns, the number of GCPs used in the adjustment was greater than four, and better results were obtained.

### 4.2 GCP spacing of 150 m

For the distance of 150 m, an improvement was observed in the results in comparison with the previous distance. For this distance interval, the highest RMSE are located in the zigzagging distribution (D-4) in scenarios E−3 and E−4 over those used for BA four and five GCPs, respectively. The offset distribution used in this study partly coincides with the work done by Skarlatos et al. [69], in which they distributed two GCPs at each end of a 2.2 km corridor and several GCPs along it, with separation distances of between 200 and 150 m. The main conclusion was that as the number of GCPs increases, accuracy improves.

When the offset-type pattern (D-5 and D-6) was followed in scenarios E−2, E−3 and E−4, the results improved substantially, reducing planimetric and, especially, altimetric errors. For these cases, the number of GCPs used in the adjustment varied between six and 10.

In E−2, the RMSE_XY_ for D-5 and D-6 cases were similar in single and double strips, with values between 3.8 and 4.5 cm. The altimetric errors were higher than for the planimetric component, with values for the single strips of between 6.1 and 6.8 cm and for the double strips of between 5.6 and 6.5 cm.

These values are similar to those found in the study by Perez et al. [48], although in that work, the authors distributed the chains between three to six GCPs every 150 m over a working area of uniform dimensions. These data indicate better behaviour of the results obtained in the double strips compared to the single strips.

For the D-4 case with a zigzagging distribution, the results obtained in E−3 show planimetric errors of between 3.4 and 6.5 cm (CSS, NSS) and 3.3 and 5.2 cm (CDS and NDS) and altimetric errors of 9.3–13.1 cm (CSS, NSS) and from 8.8 to 9.7 cm.

Regarding the cases with an offset-type GCP distribution, the RMSE_XY_ for D-5 and D-6 were obtained for single and double strips from 3.4 to 5.0 cm, and the altimetric accuracies for double strips again improve when compared with the single strips. Thus, for double strips, the RMSE_Z_ are between 5.9 and 9.0 cm, and for single strips, they are between 6.6 and 10.1 cm.

The last of the scenarios (E−4) in which the accuracies of the 150 m distance were evaluated also present similar results.

The lowest accuracies were obtained in case D-4, with a total of five GCPs used in the adjustment, reaching planimetric errors of between 4.1 and 7.5 cm and altimetric errors of 7.2–28 cm, both in single and double strips. Cases D-5 and D-6, in which eight and 10 GCPs were used, achieved better accuracy in both planimetry and altimetry. The RMSE_XY_ achieved in the four strips were similar and were between 4.1 and 5.2 cm. In the RMSE_Z_ a decreasing tendency was observed in the double strips, with values between 6.6 and 7.3 cm, and for the single strips, values between 7.6 and 9.0 cm. Also in this scenario, the altimetry results obtained in the D-6 case are slightly more precise than for D-5.

### 4.3 GCP spacing of 100 m

With an interval between GCPs of 100 m (cases D-7, D-8 and D-9) and similar patterns, there were greater errors in the zigzag type configuration (D-7) than that of the offset type (D-8 and D-9), but in these cases, the differences between the values are minor. For this GCP distribution space, the number of points used varied between five and 14 GCPs in the cases studied. The results obtained by Tokin [68] for identical separation distances were quantified in the decimetre range, as were the results obtained in this work in different scenarios in the D-7 configuration.

For this interval in E−1, planimetric errors of between 3.5 and 4 cm and altimetric errors of 4.6–5.6 cm were obtained in all strips. For the second of the scenarios (E−2), the RMSE_XY_ values obtained for the D-7 case varied between 4.3 and 5.0 cm and the RMSE_Z_ from 4.2 to 8.6 cm for single and double strips. For the other two cases (D-8 and D-9), the planimetric errors were between 3.9 cm and 5.1 cm and the altimetric errors were between 3.8 and 6.2 cm in all strips. For these last two cases, there is an improvement in accuracy when the altimetric results of the double strips are compared with the single ones, which reach an average of 1.5 cm.

Case D-9 presents slightly lower altimetric errors than D-8. The results obtained in E−3 show the same trend as that for cases D-7, D-8 and D-9, in which five, eight and 10 GCPs were used, respectively, for the adjustment of the block.

The worst results were observed in D-7, in which planimetric residual values were between 3.3 and 5.1 cm and altimetric values were 6.0–10.2 cm in the four strips.

In cases D-8 and D-9, RMSE_XY_ between 3.1 and 4.9 cm and RMSE_Z_ between 3.7 and 6.0 cm were also obtained in the four strips. For these two cases, it is also true that the results obtained with double strips were more precise than with single strips and that case D-9 presented altimetric errors slightly lower than those of case D-8.

In the last of the E−4 scenarios in which seven (D-7), 11 (D-8) and 14 (D-9) GCPs were used for each case, the results obtained were for the zigzagging distribution (D-7) in planimetry, in which some errors of 5–6.4 cm and of 6.3–8.6 cm in altimetry were found. For the other two cases with an offset-type pattern, the planimetric errors varied between 3.9 and 4.8 cm and the altimetric errors from 4.9 to 6.2 cm in the four strips.

As mentioned, the double strips present better results than the single strips, as does case D-9 compared to D-8. Similar findings were observed in work related to higher image overlap as in the cases of double strips in which the lateral overlap of images is higher. Haala et al. [70] pointed out that the combination of multiple image accuracies with large overlap increases the accuracy in 3D point clouds, and even more importantly, the roundness allows a very efficient elimination of erroneous matches, leading to higher reliability in data capture. Forlani et al. [49] showed that the accuracy and precision of the DSMs generated by UAV-SfM depend on several parameters, one of which is the overlap between strips. Also, some studies [71,72] have found that increasing the degree of overlap between images leads to an increase in geometric pressure in 3D models, but this is better when oblique images with nadir are integrated.

### 4.4 GCP spacing of 50 m

In the case of 50 m, the trends in the separation intervals between the GCPs are similar to those previously described, but in these cases, the number of GCPs used in the different cases has increased due to the lower distance; therefore, the differences found between the configurations are very small.

At a flight height of 100 m, using 18 GCPs and similar separation distances between GCPs, James et al. [51] suggested that DSM accuracies of 5 cm, similar to those obtained in this work, can be achieved. Bolkas [54] concurred on the results obtained in the assessment of accuracy for a separation interval between 85 and 65 m. They emphasised that at least 8 to 12 GCPs were needed to reduce the RMSE values to the level of 1–2 cm in less complex areas; in complex areas, between 3 and 5 cm; and 7–10 cm in those areas of greatest complexity.

In cases in which a zigzag pattern (D-10) was used, the RMSE reached in the four scenarios were slightly higher than when using the offset-type pattern (D-11 and D-12), presenting minimal differences between the planimetric and the altimetric components.

For the three cases (D-10, D-11 and D12) of scenario E−1, the RMSE_XY_ obtained were quite similar, with values between 3.0 and 4.0 cm in all strips. Nevertheless, for altimetric errors, there were differences among cases.

For D-10, the values in all the strips varied from 5.3 to 6.2 cm, and for cases D-11 and D-12, the variation was between 3.0 and 4.5 cm. For these last two cases, a decrease in accuracy can be seen in single strips compared with double strips. E−2 presents results that are similar to E−1, with RMSE_XY_ between 3.3 and 3.8 cm for the three cases and the four strips, RMSE_Z_ between 3.9 and 6.3 cm for the D-10 case and between 3.4 and 5.5 cm for D-11 and D-12. Once again, better accuracy is observed in the cases with offset distributions compared with the zigzagging type as well as in the reduction of errors between the D-11 and D-12 cases and between the single and double strips.

For the third scenario, the RMSE_XY_ were between 2.9 and 4.7 cm in the three cases and four strips, the altimetric errors in the four strips had values between 4.5 and 5.7 cm for the D-10 case and values between 2.5 and 5.2 cm for D-11 and D-12.

Once again, a loss of accuracy was observed in the configuration of case D-10 (zigzag) compared to D-11 and D12 (offset) as well as between these two cases, and an improvement was observed in the accuracy of the results obtained in D-12 compared with those of D-11.

As regards the last of the E−4 work zone, the results tended to behave similarly to the rest of the scenarios studied. The RMSE_XY_ for the four strips and three cases ranged from 3.7 to 4.7 cm; for altimetry, it ranged between 3.7 and 5.6 cm.

### 4.5 Final discussion, limitations and future lines of research

The results of this work confirm that the use of a small number of GCPs in the adjustment of the block of images produces the appearance of quite large RMSEs in the products obtained, particularly so in the altimetric part. These systematic errors affecting the vertical component, called ‘doming’, appear particularly in corridor mapping work [54] due to the accumulation of errors in camera calibration [73]. In this last work, the authors suggested that one of the strategies to mitigate this effect would be to densify the GCP distribution, reducing the distance interval.

As indicated in Ref. [54], a low number of GCPs distributed over the object studied causes an erroneous self-calibration process during the block adjustment and, therefore, poor accuracy in the generation of the DSM derived from the use of UAV. This study assessed the impact of the number and separation distance between GCPs on the elevation accuracy of UAS surveys, obtaining results indicating that at least eight to 12 GCPs are required with a separation distance of between 65 and 85 m in order to achieve the correct georeferencing and self-calibration of the camera for the work area. However, the type of distribution was not mentioned in the work.

The results obtained in our study largely coincide with those presented in the aforementioned work [54]. In all scenarios in our work, the best results were achieved when working with a separation between GCPs of 50 and 100 m. Nevertheless, it is necessary to differentiate between the RMSE_XY_ and the RMSE_Z_ obtained for the different scenarios and their distributions.

The RMSE_XY_ obtained in most cases when four GCPs were used in the BA present values between 8 and 5 cm, with the largest errors appearing in the GCP distributions with a distance interval of 200 to 150 m and the smallest between 100 and 50 m. When the number of GCPs is increased to five, planimetric errors improve to values of 6.5 to 3.5 cm, with the largest errors distributed between the GCP interval between 200 and 100 m and the smallest between 100 and 50 m. By introducing six or more control points, the RMSE_XY_ converge to mean values of 4 cm in most cases, and hardly any differences from this number of GCPs appear among the different distributions. These results are partially in agreement with those obtained in Ref. [61].

This work indicates that an increase in the number of GCPs improves planimetric accuracy and that the best horizontal accuracy was achieved when five or 10 GCPs were used in work areas with flatter morphology.

In another investigation carried out by Yu et al. [62] to determine the optimal number of GCPs in different work areas according to size, it was concluded that in small areas, three or more GCPs had to be used, while in medium and large areas, a minimum of six were needed.

If we extrapolate the results obtained in this study and equate the dimensions of the four scenarios (E−1, E−2, E−3 and E−4) to the medium and large areas of [62], we arrive at significantly similar results with regard to the number of GCPs, which is six or more.

**Fig. 13:**
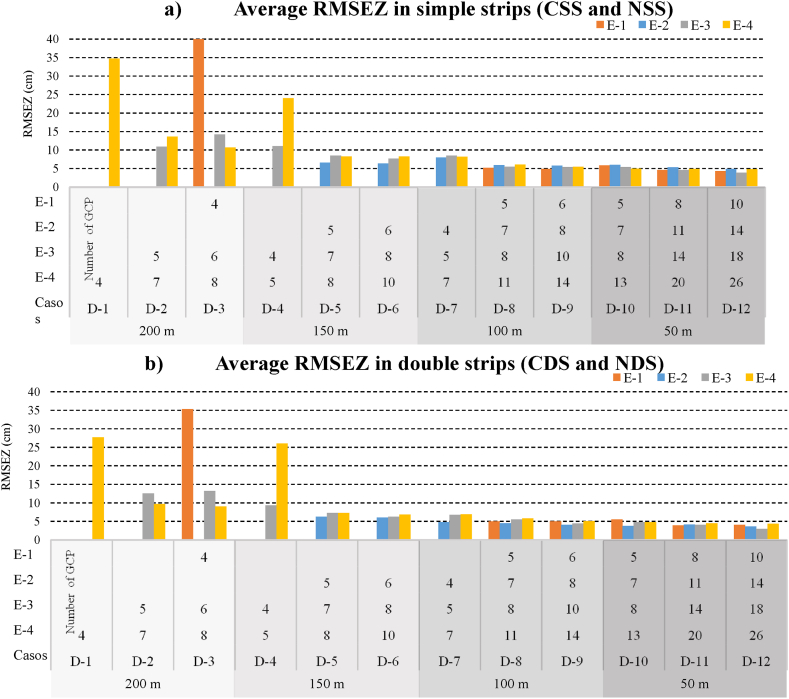
Average RMSE_Z_ based on the number of GCPs per scenario and cases with distance interval between GCPs for autonomous (a) simple flights (CSS and NSS), and, (b) for double flights (CDS and NDS).

Regarding the RMSEZ, it was considered opportune to create Table 8 and Fig. 13a and b, in which the means of the altimetric errors (Table 3, Table 4, Table 5, Table 6) obtained for single (CSS and NSS) and double strips (CDS, NDS) are presented depending on the number of GCPs used in the adjustment for each scenario (E−1, E−2, E−3 and E−4) and for each case (D-1, D-2 and D-12).

**Table 8:** Number of GCPs and average RMSE_Z_ in single (CSS, NSS) and double (CDS and NDS) strips for each scenario and case.

Configuration	GCP per scenario				RMSE_Z_ (CSS, NSS)				RMSE_Z_ (CDS, NDS)			
GCP	E4	E3	E2	E1	E1	E2	E3	E4	E1	E2	E3	E4
**D-1**	4							34.7				27.7
**D-2**	7	5					10.9	13.6			12.5	9.7
**D-3**	8	6		4	42.1		14.2	10.7	35.3		13.2	9.0
**D-4**	5	4					11.0	24.0			9.3	26.1
**D-5**	8	7	5			6.6	8.5	8.3		6.2	7.3	7.3
**D-6**	10	8	6			6.3	7.7	8.3		6.0	6.2	6.8
**D-7**	7	5	4			7.9	8.5	8.2		4.8	6.8	6.9
**D-8**	11	8	7	5	5.2	5.9	5.5	6.0	5.0	4.5	5.6	5.8
**D-9**	14	10	8	6	4.9	5.8	5.4	5.5	5.0	4.0	4.5	5.1
**D-10**	13	8	7	5	5.9	6.0	5.4	5.0	5.5	3.8	4.8	4.8
**D-11**	20	14	11	8	4.6	5.3	4.6	4.9	3.9	4.2	4.1	4.5
**D-12**	26	18	14	10	4.3	4.9	3.9	4.8	4.1	3.7	3.0	4.4

When the data in Table 8 and Fig. 13a and b are analysed, a reduction is observed in altimetric errors because the distance separating the GCPs decreases, both in single and double strips. It can thus be deduced that the best accuracies are found between separating distances of 100 and 50 m, coinciding with the study [54], in which it was stated that at least eight to 12 GCPs are needed to reduce errors, with an average separation distance of between 65 and 85 m.

In the work carried out by Tournadre et al. [74], the distribution of the GCPs in linear geometries was studied with the aim of reducing most of the re-projection errors of the projection centres and, therefore, those of the derived photogrammetric products. They concluded that one GCP per 100 m is the optimal distribution to reduce most errors, but they did not mention the type of distribution of the GCPs.

The study by Sanz-Ablanedo et al. [52] showed that the distribution of GCPs affects the accuracy of the products obtained by UAV-SfM and that a good geometric distribution of the GCPs will reduce the errors produced by the doming effect [51] and improve accuracy.

Likewise, research carried out in corridors by Ferrer-González et al. [56] found that of the different configurations tested, the projects in which they were distributed on alternate sides of the highway with an offset-type pattern yielded better results than projects that used the same or a higher number of GCPs but were otherwise distributed.

The results obtained in this work are in line with those of the previously mentioned authors [52,54,56,74] and serve to verify that the best results were obtained when the offset-type distribution was used in pairs along the road, such as cases D-6, D-9 and D-12.

No significant differences were found in the accuracy of the results obtained using nadiral or convergent measurements, as reflected in Ref. [43], noting that near-vertical imaging flights should be avoided when used for BA with UAV-SfM. Nevertheless, if the number of GCPs and their distribution is not suitable, the use of oblique or convergent shots in the UAV-SfM workflow reduces systematic errors within the resulting models [44].

As for the use of single or double strips, the improvement in accuracy from using double strips instead of single strips has also been verified in this work, although in some cases, this improvement is insignificant.

Errors, especially altimetric ones, vary depending on the type of distribution, number and distance between GCPs used in the BA, reaching optimal accuracy for separation distances of between 50 and 100 m, an offset-type distribution and a minimum number of between 6 and 100 m. Within these assumptions, the altimetric errors range in value from less than 6 cm for intervals of 100 m and less than 5 cm for distances of 50 m. These results reveal quite precise solutions, taking into account that the horizontal and vertical measurement accuracies are 1–2 cm and 2–3 cm, respectively. The results obtained in this work are encouraging in terms of the potential to improve data capture in accident reconstructions, but the methodology presented is subject to several limitations regarding the operability of UAVs.

One of these limitations arises when lighting conditions are adverse and degrade the quality of the 3D models, or when the work area is located within a difficult flight area, such as a tunnel or dense barrier (wooded area), and with various weather factors, such as temperature, wind, fog, rain or snow. These restrictive situations for flight tend to be a fundamental challenge in UAV flight missions, showing an inability to autonomously navigate in different environments. Nevertheless, continuous advancement and innovation in UAV technology is paving the way to address some of these limitations. For example, the sensors used in the cameras of this equipment are in continuous development and evolution, improving efficiency and precision through multispectral sensors and higher resolutions, with algorithms that suppress image noise, improve image quality and allow work to proceed in low light conditions. Another aspect in which this equipment is being developed is the ability to withstand high winds, and some newer UAVs [75] can even be used effectively in heavy rain, high winds and at high altitudes, even in icy and snowy conditions.

Another possible limitation on the application of the approaches to accident reconstruction presented in this work relates to the implementation of the proposed methodology by police forces, as it implies specific technical knowledge for the use and handling of the procedures and tools. The requirements for qualified personnel could lead to an increase in costs and, therefore, make this proposal less beneficial. However, the technical requirements necessary to carry out all the work described in this paper could be achieved with basic training, which requires little investment. In addition, all operations could be executed by a single agent, which would reduce the cost.

Likewise, material prices could be expected to increase as the demand for materials increases or production decreases. However, technological advances usually lead to an increase in the cost of materials due to the incorporation of more features. Therefore, the tools used in this study will maintain their cost or even decrease in the future. In future research it would be particularly interesting to use post-processing kinematic (PPK) or real-time kinematic (RTK) solutions to reduce the time dedicated to the capture of GCPs. Nevertheless, RTK and PPK methods require expensive devices and complex technologies that make their use and the incorporation of these sensors to UAVs difficult as they involve an increase in the weight of the MTOW and the forfeiture of the C0 class marking and, along with it, the loss of the advantages and ease of use of this type of device.

Another future line of work would be to implement flights with double strips at different flight altitudes, combining nadir and convergent shots for each of the strips to avoid possible systematic errors and to try to reduce the number and separation between GCPs.

## 5 Conclusions

In this work, we examined the geometric quality of the different products obtained by UAV-SfM (orthomosaic, point cloud and DSM) using a set of procedures and a combination of low-cost UAS and GNSS equipment to reconstruct large and small traffic accident scenes. The use of any other software and hardware with identical technical characteristics to those described in this work would lead to similar results.

The main aim of the research was to develop a work methodology involving economical tools that could be applied by any police reconstruction team to conduct efficient capture, measurement, processing and generation of 3D models in a short time from an absolute position and with sufficient accuracy to generate a complete 3D reconstruction of° the area of the accident and to do so without excessively interfering with the accident scene.

For this task, the reconstruction of four linear scenarios of simulated accidents with different configurations and numbers and separation intervals between the GCPs was carried out through manual and autonomous flights in simple and double strips and photographic captures with nadiral and convergent geometry.

For each of the scenarios with their corresponding case studies, both horizontal and vertical accuracy improved by decreasing the separation distance between GCPs and the use of an offset-type distribution pattern, thus increasing the number of GCPs used in the BA. Better accuracy was achieved in planimetry over altimetry throughout.

The best results were achieved in those offset-type distributions in which the GCPs were located on both sides of the work area and at each of the ends with a separation of between 100 and 50 m. Average RMSE values from 4 to 3.5 cm were obtained for the horizontal plane and from 6 to 5 cm for the vertical plane.

It was also evident in this work that results were improved by making flights with double strips instead of single strips.

Thus, we consider that the procedure to achieve optimal results would be:

⁃ Distribution of the GCPs in an offset pattern located on both sides of the work area and with a separation interval of between 100 and 50 m. The ideal would be to use an average separation between these distances to obtain intermediate accuracy values of between 6 and 5 cm. For example, the optimal distance to use would be between 85 and 65 m, so that in scenarios of reduced length, the total number of GCPs would be at least eight with the offset-type pattern.
⁃ Use of various PCs to be able to make checks *a posteriori* on the different outputs obtained by UAV-SfM.
⁃ Execution of the flight, preferably autonomously, with double flight lines or double strips, using frontal overlaps of between 80 % and 85 %, and 85 % for the sides.

Based on the results obtained in the different tests in this research, the benefits of the combined use of UAS and low-cost GNSS receivers for digital reconstruction of large and small accident scenes have been highlighted. This methodology allows full 3D captures of the work area in great detail, in a simple, fast, reliable, precise way, and at a low cost for its generalised usage.

## Funding statement

This research did not receive any specific grants from funding agencies in the public, commercial, or non-profit sectors.

## Data availability statement

The data associated with our work has not been deposited into a publicly available repository. The data will be available on request.

## Additional information

No additional information is available for this paper.

## CRediT authorship contribution statement

**Juan Antonio Pérez:** Writing – review & editing, Writing – original draft, Validation, Supervision, Methodology, Investigation, Formal analysis, Data curation, Conceptualization. **Gil Rito Gonçalves:** Writing – review & editing, Writing – original draft, Validation, Methodology, Investigation, Formal analysis, Data curation, Conceptualization. **Juan Ramón Morillo Barragan:** Writing – review & editing, Writing – original draft, Validation, Investigation, Formal analysis, Data curation, Conceptualization. **Pedro Fuentes Ortega:** Writing – review & editing, Writing – original draft, Validation, Methodology, Investigation, Formal analysis, Data curation, Conceptualization. **Antonio Antonio M. Caracol Palomo:** Writing – review & editing, Writing – original draft, Validation, Methodology, Investigation, Formal analysis, Data curation, Conceptualization.

## Declaration of competing interest

The authors declare that they have no known competing financial interests or personal relationships that could have appeared to influence the work reported in this paper.
